# Blockade of DDR1/PYK2/ERK signaling suggesting SH2 superbinder as a novel autophagy inhibitor for pancreatic cancer

**DOI:** 10.1038/s41419-023-06344-4

**Published:** 2023-12-09

**Authors:** Hui Xu, Ming Tan, Guo-Qing Hou, Ya-Zhou Sang, Li Lin, Xiao-Cai Gan, Xuan Cao, An-Dong Liu

**Affiliations:** 1 School of Basic Medicine, Tongji Medical College, Huazhong University of Science and Technology, 430030 Wuhan, Hubei China; 2 School of Medicine, Taizhou University, 318000 Taizhou, Zhejiang China; 3 Wenling First People’s Hospital (The Affiliated Wenling Hospital of Taizhou University), School of Medicine, Taizhou University, 318000 Taizhou, Zhejiang China; 4 National Demonstration Center for Experimental Basic Medical Education, Huazhong University of Science and Technology, 430030 Wuhan, Hubei China

**Keywords:** Tumour-suppressor proteins, Cancer therapy

## Abstract

Pancreatic cancer is highly lethal, of which 90% is pancreatic ductal adenocarcinoma (PDAC), with a 5-year survival rate of less than 12%, lacking effective treatment options and late diagnosis. Furthermore, the tumors show an intense resistance to cytotoxic chemotherapies. As autophagy is elevated in PDAC, targeting the autophagic pathway is regarded as a promising strategy for cancer treatment. Immunofluorescence and transmission electron microscopy were utilized to assess the autophagic flux. Label-free quantitative phosphoproteomics was used to figure out critically altered tyrosine phosphorylation of the proteins. Tumor-bearing mice were used to validate that SH2 TrM-(Arg)9 restrained the growth of tumor cells. SH2 TrM-(Arg)9 inhibited collagen-induced autophagy via blocking the DDR1/PYK2/ERK signaling cascades. SH2 TrM-(Arg)9 improved the sensitivity of PANC-1/GEM cells to gemcitabine (GEM). Inhibition of autophagy by SH2 TrM-(Arg)9 may synergized with chemotherapy and robusted tumor suppression in pancreatic cancer xenografts. SH2 TrM-(Arg)9 could enter into PDAC cells and blockade autophagy through inhibiting DDR1/PYK2/ERK signaling and may be a new treatment strategy for targeted therapy of PDAC.

## Introduction

Pancreatic cancer (PC) is a highly fatal malignancy in the digestive system, threatening hundreds of thousands of lives worldwide with a 5-year overall survival rate of only 12% [1]. Approximately 90% of pancreatic malignancies are pancreatic ductal adenocarcinomas (PDAC) originated in the pancreatic ductal epithelium [1, 2]. Many patients with PC are already in the middle or advanced stages when diagnosed due to the unique location and function of the pancreas; therefore, surgical treatment alone is not effective. Chemotherapy is usually the mainstay in treating advanced PDAC. Gemcitabine (GEM), combined with paclitaxel or FOLFIRINOX (fluorouracil, irinotecan, leucovorin, and oxaliplatin), is currently the first-line chemotherapy strategy [3]. However, the effectiveness of chemotherapy for advanced PDAC is limited due to chemoresistance and side effects [2]. Other treatment options, such as targeted therapy and immunotherapy, slow down the development of PDAC to a certain extent, but there is no substantial advantage over chemotherapy [4, 5]. Additionally, some of these treatment options only benefit a small group of patients with specific genotype [3]. Therefore, it is necessary to find new therapeutics with higher efficacy, wider beneficiaries, and less drug resistance.

Autophagy is a self-conserving process in which cells recycle biomolecules and damage organelles under stress or starvation conditions. Autophagy produces intermediate metabolites for biosynthesis and energy production, and promotes tumor growth by degrading and recycling damaged DNA, misfolded proteins, and intracellular pathogens [6]. Abnormal activation of autophagy flux exists in PDAC tumor tissues, cancer cells, and stem cells [7, 8]. Autophagy of other cells in the PDAC tumor microenvironment (such as pancreatic stellate cells) also provides energy for tumor growth and development [9]. Many studies have shown that the increased level of autophagy may be an essential cause of PDAC development [10, 11]. Therefore, inhibition of autophagy is thought to be an important strategy to treat PDAC. To date, small-molecule drugs that inhibit autophagy and novel chelators have shown sound anticancer effects in vitro and in vivo [12, 13].

The SH2 domain is a conserved sequence that can specifically bind to phosphorylated tyrosine (pY), and regulate various cellular processes by affecting the interaction between proteins [14]. Abnormal pY levels of proteins play an important role in the cancer development [15, 16]. The SH2 superbinder, a triple mutant of the SH2 domain, has a stronger binding ability for pY than the wild-type SH2 domain [17]. A previous study has proved that SH2 superbinder can capture a variety of pY proteins, blockade multiple signaling pathways, and suppress the growth of PDAC cells [18]. Therefore, the SH2 superbinder (termed SH2 TrM-(Arg)9 in the following contents) is expected to be a candidate drug for the treatment of PDAC. Meanwhile, we found that the SH2 superbinder could inhibit the autophagy of PDAC cells, but the precise mechanism remained undisclosed.

The collagen receptor DDR1 (discoidin domain receptor 1) is a member of the receptor tyrosine kinase (RTK) family which mediates the migration and proliferation of several cell types [19]. DDR1 displays sustained activation upon interaction with collagen and the high expression level of DDR1 is significantly correlated with poor PDAC survival, implying that collagen/DDR1 mediated signaling would play an important role in cancer progression [20]. However, the relationship between DDR1 and autophagy has been poorly reported.

In this study, we uncovered a mechanism driven by DDR1/PYK2/ERK signaling that controls PDAC therapy resistance, expecting to provide new research ideas and treatment strategies for targeted therapy of PDAC.

## Results

### SH2 TrM-(Arg)9 attenuated cell viability and induced apoptosis of PDAC cells

**Fig. 1:**
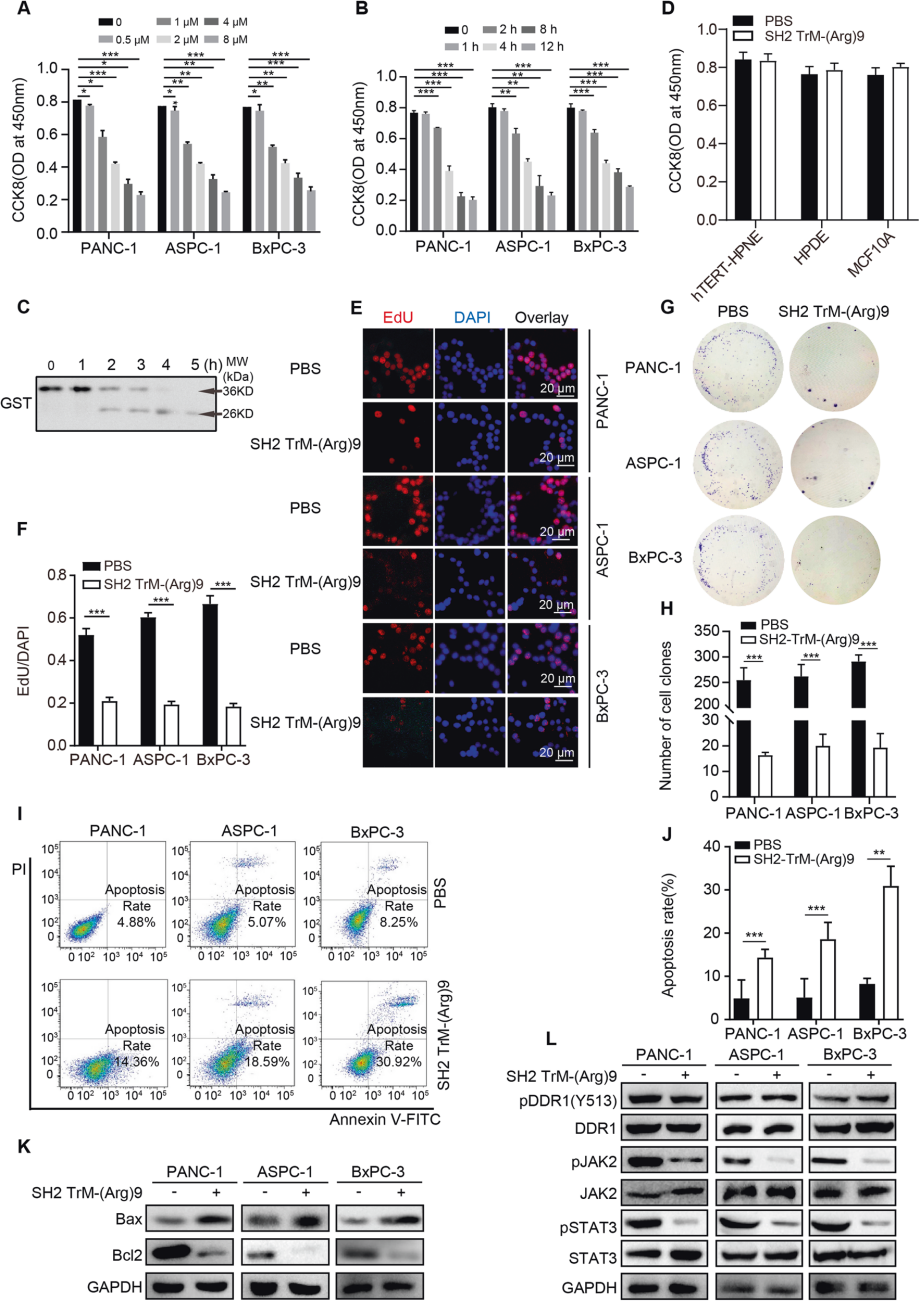
SH2 TrM-(Arg)9 attenuated cell viability and induced apoptosis of pancreatic cells. **A**, **B** Effects of SH2 TrM-(Arg)9 on the proliferation of PANC-1, ASPC-1, and BxPC-3 cells. Cells were treated with SH2 TrM-(Arg)9 at different incubation concentrations (**A**) or periods (**B**). Cell viability was measured by CCK-8 assays. **C** Degradation of SH2 TrM-(Arg)9 with anti-GST. **D** Effects of SH2 TrM-(Arg)9 on the proliferation of hTERT-HPNE, HPDE and MCF10A (*n* = 3). **E**, **F** Changes in cell proliferation between PDAC cells after SH2 TrM-(Arg)9 incubation were determined by EdU assay (*n* = 3). **G** Representative images of colony formation assay showing colonies formed by cells incubated with SH2 TrM-(Arg)9. **H** Bar graph depicting the change in the number of cell colonies (*n* = 3). **I** Flow cytometry measurement of cell apoptosis induced by SH2 TrM-(Arg)9 at 1 μM for 2 h. **J** Bar graph depicting the change in the rate of apoptosis cells (*n* = 3). **K** Western blot of Bax and Bcl2 in PDAC cells after SH2 TrM-(Arg)9 incubation. **L** Western blot of pDDR1(Y513), DDR1, pJAK2, JAK2, pSTAT3 and STAT3 in PDAC cells after SH2 TrM-(Arg)9 treatment. The data shown are representative of three independent experiments. ^*^P < 0.05, ^**^P < 0.01, ^***^P < 0.001.

To investigate the cell proliferation and cytotoxicity effects of SH2 TrM-(Arg)9, CCK-8 assays were conducted on PANC-1, ASPC-1 and BxPC-3 cells. The results showed SH2 TrM-(Arg)9 decreased cell viability in a dose- and time-dependent manners (Fig. 1A, B). Meanwhile, SH2 TrM-(Arg)9 protein was degraded in cell culture medium after 4 h (Fig. 1C). Considering data above, we chose the usage condition (1 µM, 2 h) of SH2 TrM-(Arg)9 in the following assays.

SH2 TrM-(Arg)9 showed stronger inhibition effects on proliferation, apoptosis and pY-mediated signaling pathways activation, whereas SH2 WT-(Arg)9 showed no obvious cytotoxicity (Fig. S1A–H). To further investigate whether SH2 TrM-(Arg)9 could affect non-cancerous cells, we cultured SH2 TrM-(Arg)9 or SH2 WT-(Arg)9 with hTERT-HPNE (immortalized pancreatic cells), HPDE (human normal pancreatic ductal epithelial cells), and MCF10A (human mammary epithelial cell line) cells. The results exhibited SH2 TrM-(Arg)9 did not affect the survival of hTERT-HPNE, HPDE, or MCF10A cells (Fig. 1D). EdU assays reconfirmed SH2 TrM-(Arg)9 attenuated cell viability (Fig. 1E, F). Next, colony formation assays were performed to identify whether SH2 TrM-(Arg)9 could affect the long-term colony formation of PDAC cells. The results demonstrated SH2 TrM-(Arg)9 significantly reduced the number of surviving colonies (Fig. 1G, H). To validate whether SH2 TrM-(Arg)9-induced cell death was caused by apoptosis, the FITC-labeled Annexin V/PI staining and flow cytometry were used. Cells treated with SH2 TrM-(Arg)9 underwent apoptosis (Fig. 1I, J). The expression level of the pro-apoptotic protein Bax was increased, and the anti-apoptotic protein Bcl2 was decreased (Fig. 1K).

As the DDR1/JAK/STAT signaling pathways are involved in cancer progression [21], we evaluated whether the vital proteins would be affected by SH2 TrM-(Arg)9. Up to now, DDR1 has gotten less attention compared to other RTKs, such as EGFR, VEGFR et al. Besides, in the existing researches, Y513 of DDR1 is critical for the DDR1 mediated signaling, which functions involved cell migration, invasion, stem cell differentiation and et al. [22–25]. Y792 is another phosphorylation site located in kinase domain of DDR1, which may affect the kinase activity of DDR1 [26]. But little is known about the function of this site. In this paper, we evaluated the effect of SH2 TrM-(Arg)9 on Y513 and Y792 of DDR1, and it was interesting that SH2 TrM-(Arg)9 only restrained phosphorylation of Y792, but not Y513 of DDR1 (Fig. 3). The pSTAT3 and pJAK2 were decreased after SH2 TrM-(Arg)9 treatment, whereas the DDR1(pY513) showed no obvious change (Fig. 1L). These data revealed SH2 TrM-(Arg)9 might lead to cell death progression by blocking the JAK/STAT signaling pathway in PDAC cells without affecting DDR1(Y513).

### SH2 TrM-(Arg)9 suppressed autophagy in PANC-1 Cells

**Fig. 2:**
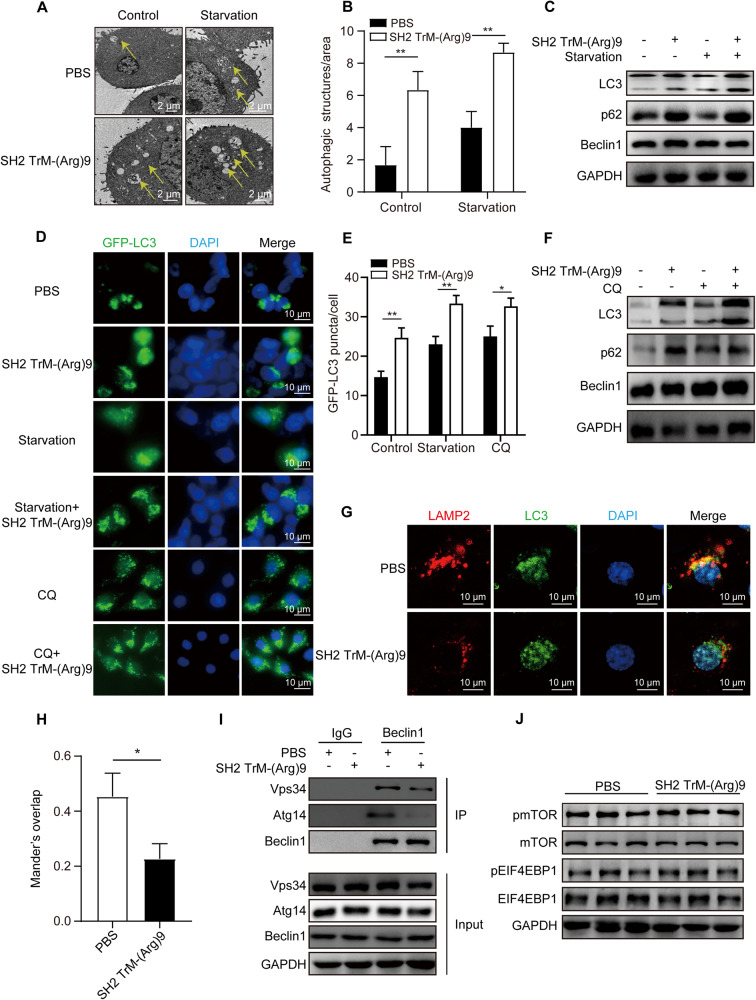
SH2 TrM-(Arg)9 suppressed autophagy in PANC-1 Cells. **A**, **B** Representative images of autophagosomes or autolysosomes of the PANC-1 cells treated with SH2 TrM-(Arg)9 in nutrient-rich or EBSS starvation conditions. Yellow arrows indicate autophagic structures. **C** Western blot of LC3, p62, and Beclin1 in PANC-1 cells after SH2 TrM-(Arg)9 incubation in nutrient-rich or EBSS starvation conditions. **D**, **E** Representative fluorescence images of GFP-LC3 in PANC-1 cells after SH2 TrM-(Arg)9 or CQ treatment in nutrient-rich or EBSS starvation conditions. **F** Western blot of LC3, p62, and Beclin1 in PANC-1 cells after SH2 TrM-(Arg)9 or CQ treatment. **G**, **H** The immunofluorescence staining of LAMP2 (lysosome-associated membrane protein 2) and LC3. **I** Co-immunoprecipitation of endogenous Beclin1 with Vps34 and ATG14 from PANC-1 whole-cell lysates (WCL) upon SH2 TrM-(Arg)9 treatment. **J** Western blot of pmTOR, mTOR, pEIF4EBP1 and EIF4EBP1 in PANC-1 cells after SH2 TrM-(Arg)9 incubation. The data shown are representative of three independent experiments.

Our previous data [17] have shown that SH2 TrM-(Arg)9 possesses anti-tumor activity and has a wide range of mechanisms. However, the relationship between SH2 TrM-(Arg)9 and autophagy is unclear. We used transmission electron microscopy to observe the autophagosomes and autolysosomes, and to investigate whether SH2 TrM-(Arg)9 would influence autophagy in PDAC cells. Consistent with previous study [27], PDAC cells showed elevated autophagy under basal conditions. As shown in Fig. 2A, B, the number of autophagosomes was markedly increased in PANC-1 cells treated with SH2 TrM-(Arg)9, but not in the control groups. Next, we analyzed the levels of LC3, p62, and Beclin1. The results showed SH2 TrM-(Arg)9 may induce the accumulation of LC3 (Fig. 2C). Meanwhile, levels of p62 increased, but Beclin1 showed no obvious changes (Fig. 2C). This observation also confirmed SH2 TrM-(Arg)9 markedly increased the number of GFP-LC3-positive puncta transiently transfected with GFP-LC3 constructs (Fig. 2D, E).

The increased levels of LC3-II or autophagic vacuoles are insufficient evidence to demonstrate autophagic flux induction, as they can be a result of an increase in their formation either from this induction or from the inhibition of autophagic degradation [28]. In this regard, we demonstrated SH2 TrM-(Arg)9 had no significant effect on the expression levels of autophagy core genes. The increased LC3-II levels after SH2 TrM-(Arg)9 treatment were further increased by chloroquine (CQ).

CQ, a clinically available antimalarial agent, blocks late-stage autophagy by impairing lysosomal acidification and has been used to inhibit autophagy in patients [29, 30]. Compared with CQ, SH2 TrM-(Arg)9 increased LC3-II to a higher level and further enhanced LC3-II levels in cells pre-treated with CQ (Fig. 2F). These data indicated that increased LC3-II levels in SH2 TrM-(Arg)9-treated cells were unlikely due to the enhanced autophagic flux but the suppression of the late maturation and degradation stages. We further used immunofluorescence staining of LAMP2 and LC3 to confirm this finding. The images displayed SH2 TrM-(Arg)9 impaired autolysosome formation (Fig. 2G, H). Meanwhile, p62 increased, and there was no change in Beclin1 after cells were stimulated with SH2 TrM-(Arg)9 (Fig. 2F). These findings suggested SH2 TrM-(Arg)9 blocked autophagic flux in a similar way to CQ and exhibited a stronger inhibitory effect on autophagy.

The induction of autophagy is closely related to the assembly of the Beclin1/Vps34/ATG14 core complex [31]. To assess the role of SH2 TrM-(Arg)9 in autophagy regulation, we employed Beclin1 immunoprecipitation and observed Beclin1 dissociation from Vps34 and Atg14 under SH2 TrM-(Arg)9 administration (Fig. 2I). The results showed SH2 TrM-(Arg)9 impaired the formation of Beclin1/Vps34/ATG14 complex.

Inhibition of the mTOR-mediated signaling pathway plays an essential role in promoting autophagy [32]. To investigate whether the mTOR-mediated signaling pathway would be affected during the SH2 TrM-(Arg)9-inhibited autophagy process, we evaluated the pmTOR and pEIF4EBP1 (the downstream effector protein of mTORC). The data implied SH2 TrM-(Arg)9 had no noticeable effect on the phosphorylation and total levels of mTOR or EIF4EBP1 in PANC-1 cells (Fig. 2J). These findings indicated SH2 TrM-(Arg)9-mediated autophagy inhibition might be independent of the mTOR signaling pathway, but a result of other signaling cascades.

### SH2 TrM-(Arg)9 mediated autophagy inhibition of PDAC cells through affecting phosphotyrosines

**Fig. 3:**
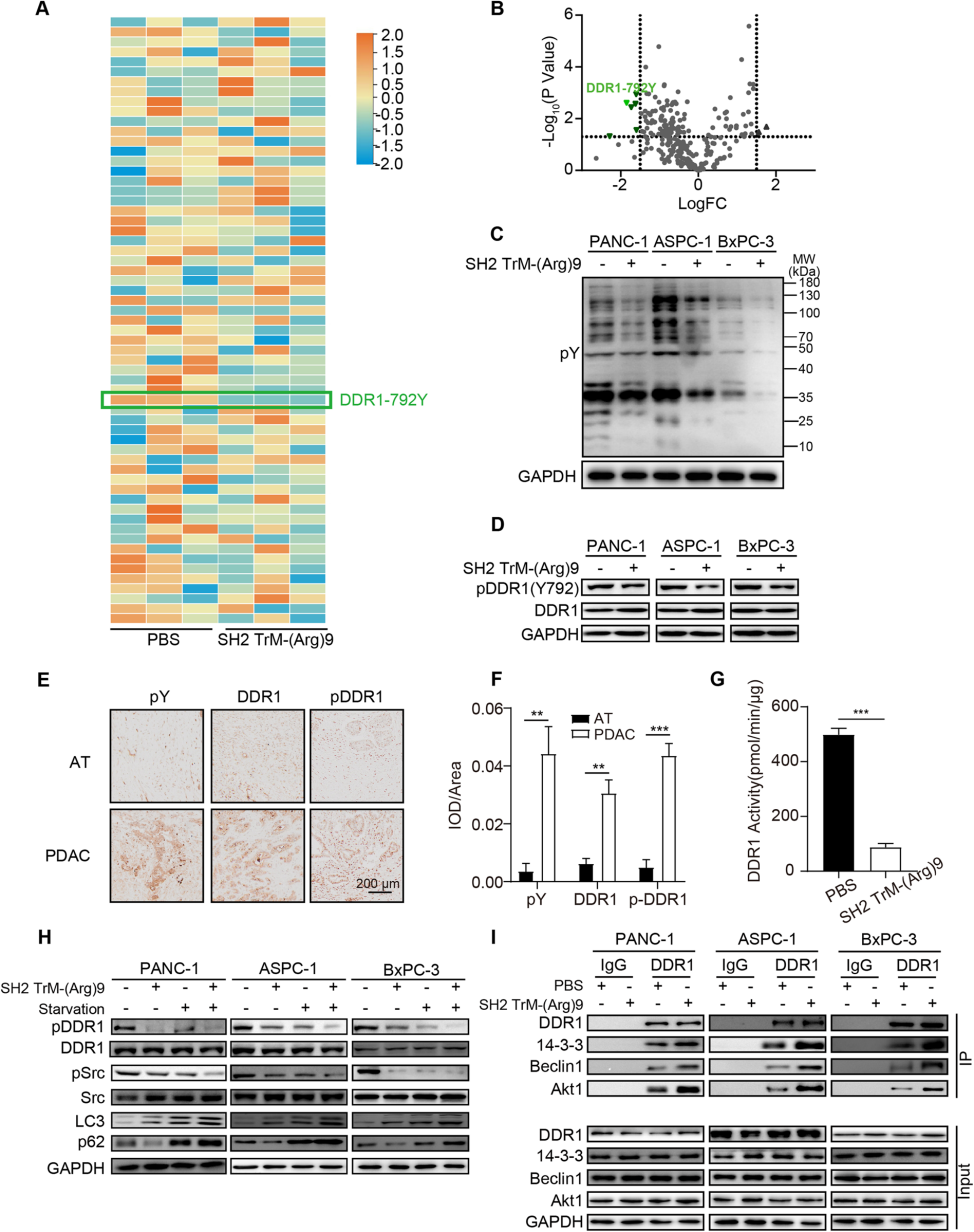
SH2 TrM-(Arg)9 mediated autophagy inhibition of PANC-1 cells through affecting phosphotyrosines. **A** The label-free quantitative phosphoproteomics was utilized to screen differential proteins. The hierarchical clustering of specific tyrosine sites with high relative phosphorylation of PANC-1 cells after SH2 TrM-(Arg)9 treatment (*n* = 3). **B** Volcano map screening for differential proteins. **C** Western blot of pY in PDAC cells after SH2 TrM-(Arg)9 incubation. **D** Western blot of pDDR1(Y792) and DDR1 in PDAC cells upon SH2 TrM-(Arg)9 treatment. **E** The orthotopic PDAC tumors and the adjacent tissues (AT) from patients with PDAC who underwent surgical resection were subject to IHC analysis with anti-pDDR1(Y792), anti-DDR1 and anti-pY. Scale bar: 200 µm. **F** Quantification immunostaining of pY, pDDR1(Y792) and DDR1 (*n* = 3). ^**^P < 0.01, ***P < 0.001. **G** DDR1 kinase (Recombinant human DDR1) activity in the presence and absence of SH2 TrM-(Arg)9 was measured by using an ADP-based phosphatase-coupled kinase assay (*n* = 3).***P < 0.001. **H** Western blot of pDDR1(Y792), DDR1, pSrc, Src, LC3 and p62 in PDAC cells. **I** Co-immunoprecipitation between DDR1 and 14-3-3-Beclin1-Akt1 complex with endogenous DDR1 from WCL of PDAC cells treated with SH2 TrM-(Arg)9 or PBS. IgG as control. Cells (**H**, **I**) under different administrations were pre-treated with collagen (40 µg/mL, 24 h). Representative western blots are shown (*n* = 3).

The aberrant activation of tyrosine phosphorylation has been reported to play an essential role in human specimens and PDAC cells [18]. To unveil the molecular mechanism underlying the physiological activities, we systematically investigated the global phosphorylation events of PANC-1 cells caused by SH2 TrM-(Arg)9 through label-free quantitative phosphoproteomics [33], expecting to figure out the critically altered tyrosine phosphorylation of proteins during the anti-tumor process. After three independent experiments, we found that Y792 of DDR1 (a member of the RTK family) showed a consistent trend of change in the three sources of PANC-1 cells, which indicated the importance of this site (Dataset 1). DDR1 plays a role in neurological disorders during the autophagy process [34, 35]. In pancreatic cancer, blocking the collagen/DDR1-based signaling pathway inhibits tumor growth [36, 37]; however, its effect on autophagy remains unknown. From phosphoproteomics results, we found the level of phosphotyrosines of DDR1(Y792) was significantly decreased in SH2 TrM-(Arg)9 treated groups (Fig. 3A, B). To further confirm the significance of the phosphotyrosines-related signaling pathways, we measured the phosphorylation levels of tyrosine (pY) and DDR1. The data indicated SH2 TrM-(Arg)9 downregulated the phosphorylation levels of tyrosine residues (Fig. 3C, D). We further measured the phosphorylation levels of total tyrosine and DDR1(Y792) in PDAC and adjacent tissues from patients. Using immunostaining, we found both pY and pDDR1(Y792) levels elevated in tumor tissues (Fig. 3E, F).

As the tyrosine 792 site is located in the kinase domain of DDR1, pDDR1(Y792) decreased due to SH2 TrM-(Arg)9 may disrupt the kinase activity of DDR1, blocking signal transduction. Intriguingly, we found the kinase activity of DDR1 had nearly 80% reduction by SH2 TrM-(Arg)9 (Fig. 3G). Src, a non-receptor kinase, which participates in cytoprotective autophagy, could co-immunoprecipitate with DDR1 and mediate tyrosine phosphorylation of DDR1 [38, 39]. The phosphorylation levels of Src were affected by SH2 TrM-(Arg)9 (Fig. 3H), indicating that SH2 TrM-(Arg)9 inhibited the DDR1-mediated signaling pathway downstream. Combining the autophagy inhibition and degradation of pSrc caused by SH2 TrM-(Arg)9 (Fig. 3H), we considered the reason was probably disruption of DDR1 kinase activity by SH2 TrM-(Arg)9.

A previous report has revealed that DDR1 interacts with the 14-3-3/Beclin1/Akt1 protein complex and regulates autophagy-mediated therapy sensitivity in glioblastoma through phosphorylation of the tyrosine 513 site [32]. In our study, we found that SH2 TrM-(Arg)9 decreased the level of pDDR1(Y792) (Fig. 3D) without affecting pDDR1(Y513) (Fig. 1L), and DDR1 bounded with Beclin1,14-3-3 and Akt1 more tightly in SH2 TrM-(Arg)9-treated PDAC cells (Fig. 3I), suggesting that SH2 TrM-(Arg)9 blocked autophagic flux through regulating the interaction between DDR1 and the 14-3-3/Beclin1/Akt1 complex. Besides, the total expression of Beclin1 was stable (Fig. 2F).

### SH2 TrM-(Arg)9 mediated autophagosome-lysosome fusion inhibition via DDR1/PYK2/ERK signaling without affecting ROS level

**Fig. 4:**
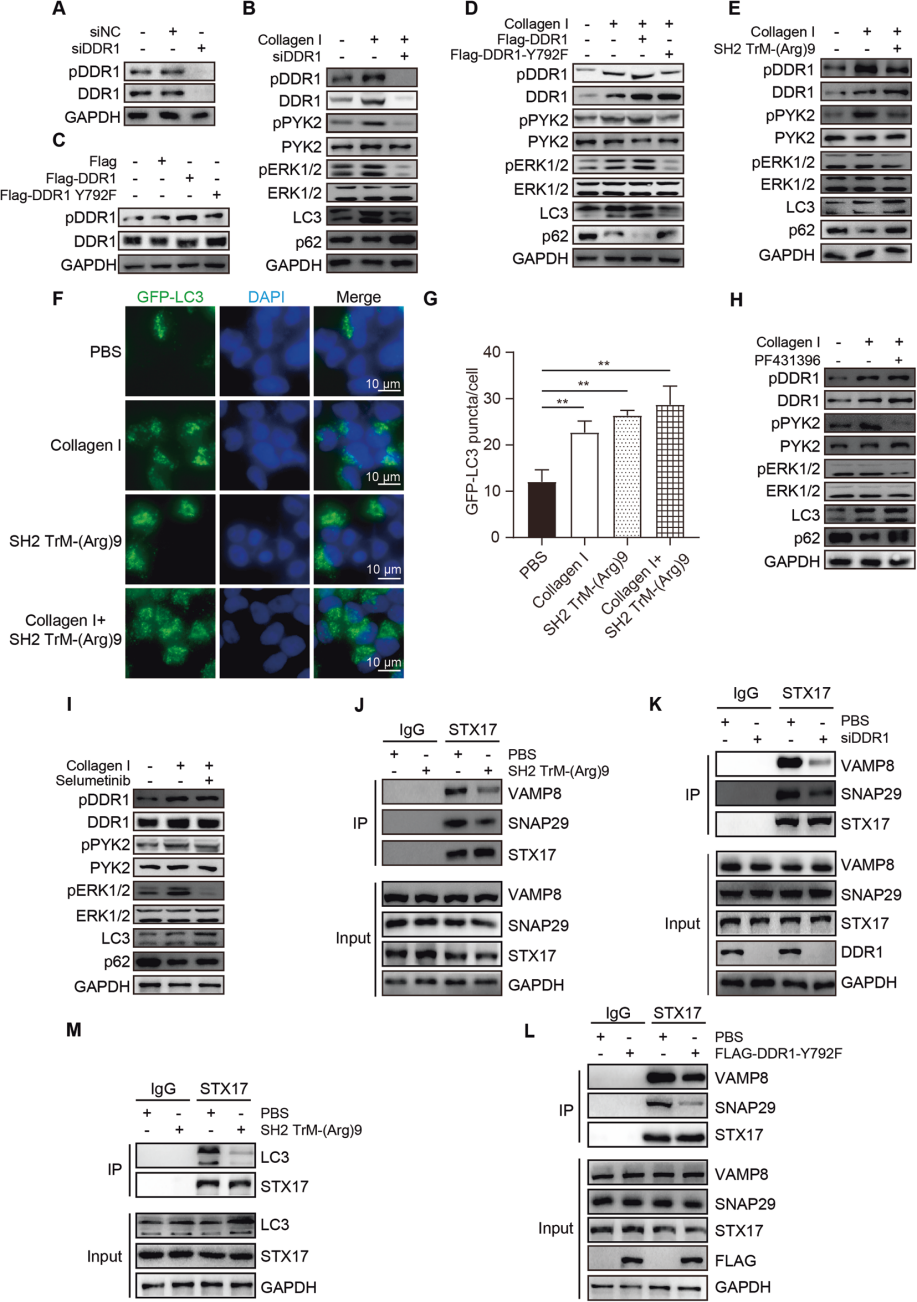
SH2 TrM-(Arg)9 mediated autophagosome-lysosome fusion inhibition via DDR1/PYK2/ERK signaling. **A** Western blot of pDDR1 and DDR1 after siDDR1administration. **B** Western blot of pDDR1, DDR1, pPYK2, PYK2, pERK1/2, ERK1/2, LC3, and p62 after treated with collagen or siDDR1. **C** Western blot of pDDR1 and DDR1 after cells were transfected with pcDNA3.1-Flag, pcDNA3.1-Flag-DDR1 (Flag-DDR1), and pcDNA3.1-Flag-DDR1 Y792F (Flag-DDR1 Y792F). **D** Western blot of pDDR1, DDR1, pPYK2, PYK2, pERK1/2, ERK1/2, LC3, and p62 after treated with collagen or transfected with pcDNA3.1-Flag-DDR1 (Flag-DDR1) and pcDNA3.1-Flag-DDR1 Y792F (Flag-DDR1 Y792F). **E** Western blot of pDDR1, DDR1, pPYK2, PYK2, pERK1/2, ERK1/2, LC3, and p62 after being treated with collagen or SH2 TrM-(Arg)9. **F**, **G** Representative fluorescence images of GFP-LC3 in PANC-1 cells after SH2 TrM-(Arg)9 or collagen treatment. **H**, **I** Western blot of pDDR1, DDR1, pPYK2, PYK2, pERK1/2, ERK1/2, LC3, and p62 after treatment with collagen or PF431396 (T2314, Targetmol, USA) and Selumetinib (T6218, Targetmol, USA). **J** Co-immunoprecipitation of endogenous STX17 with VAMP8 and SNAP29 from PANC-1 WCL upon SH2 TrM-(Arg)9 treatment. **K** Co-immunoprecipitation of endogenous STX17 with VAMP8 and SNAP29 from PANC-1 WCL upon siDDR1 treatment. **L** Co-immunoprecipitation of endogenous STX17 with VAMP8 and SNAP29 from PANC-1 WCL upon pcDNA3.1-Flag-DDR1 Y792F (Flag-DDR1 Y792F) treatment. **M** Interaction of STX17 and LC3 was affected by SH2 TrM-(Arg)9. IgG as control. Results are representative of three independent experiments with similar results.

To further clarify the precise DDR1 mediated signaling regulated by SH2 TrM-(Arg)9, PANC-1 cells were transfected with siRNA targeting DDR1 or pcDNA3.1(+)-Flag-DDR1 Y792F (Y792F) to evaluate the effect of the DDR1(Y792) on autophagosome-lysosome fusion. PYK2 and ERK1/2 are downstream effectors of collagen/DDR1, and PYK2 can activate ERK1/2 [40–42]. The results showed that collagen I could activate DDR1, PYK2 and ERK1/2, whereas siDDR1, Y792F, and SH2 TrM-(Arg)9 decreased pDDR1, pPYK2 and pERK1/2 levels (Fig. 4A-E), implying that SH2 TrM-(Arg)9 downregulated the collagen-mediated DDR1/PYK2/ERK signaling. In addition, levels of p62 and LC3 showed that SH2 TrM-(Arg)9, PF431396 (PYK2 inhibitor), and Selumetinib (ERK inhibitor) reversed the autophagy induced by collagen I (Fig. 4F–I and S2). These analyses provided insights into the cellular response mechanisms induced by SH2 TrM-(Arg)9 through the DDR1/PYK2/ERK-mediated signaling.

What is more, we also found that JAK/STAT signaling pathway had little effect on PDAC cells autophagy in our study. Altered JAK/STAT expression has been found to be accompanied by the abnormal autophagy activity in many oncological studies [43, 44]. In Fig. 1L, we have found SH2 TrM-(Arg)9 could block the JAK/STAT signaling pathway, but it is interesting that SH2 TrM-(Arg)9 not Tofacitinib (JAK/STAT inhibitor) restrain autophagy of PDAC cells (Fig. S1I). So, we considered other signaling pathway but not JAK/STAT engaged in the regulation of autophagy. All these results above inspired us that DDR1-mediated signaling pathway may regulate autophagy of PDAC cells.

Autophagosome-lysosome fusion is mediated by the soluble N-ethylmaleimide-sensitive factor attachment protein receptor (SNARE) complex, composed of STX17, which connects SNAP29 to autophagosomes and VAMP8 to lysosomes [45]. SH2 TrM-(Arg)9, siDDR1, and Y792F dramatically inhibited SNAP29/STX17/VAMP8 complex formation (Fig. 4J-L). SH2 TrM-(Arg)9 prevented STX17 association with LC3 (Fig. 4M), which abolished autophagic flux [46].

As autophagic flux is regulated by ROS/NRF2 signaling pathway [47], combined with previous results, we tested whether SH2 TrM-(Arg)9 would affect ROS. These data showed SH2 TrM-(Arg)9 did not cause any apparent changes in ROS levels (Fig. S3A, B). We also examined some pivotal proteins (KEAP1 and NRF2) (Fig. S3C). These data implied SH2 TrM-(Arg)9 inhibited autophagy without affecting the ROS/NRF2 signaling.

### SH2 TrM-(Arg)9 mediated-autophagy inhibition sensitizes PANC-1/GEM cells to gemcitabine in vitro

**Fig. 5:**
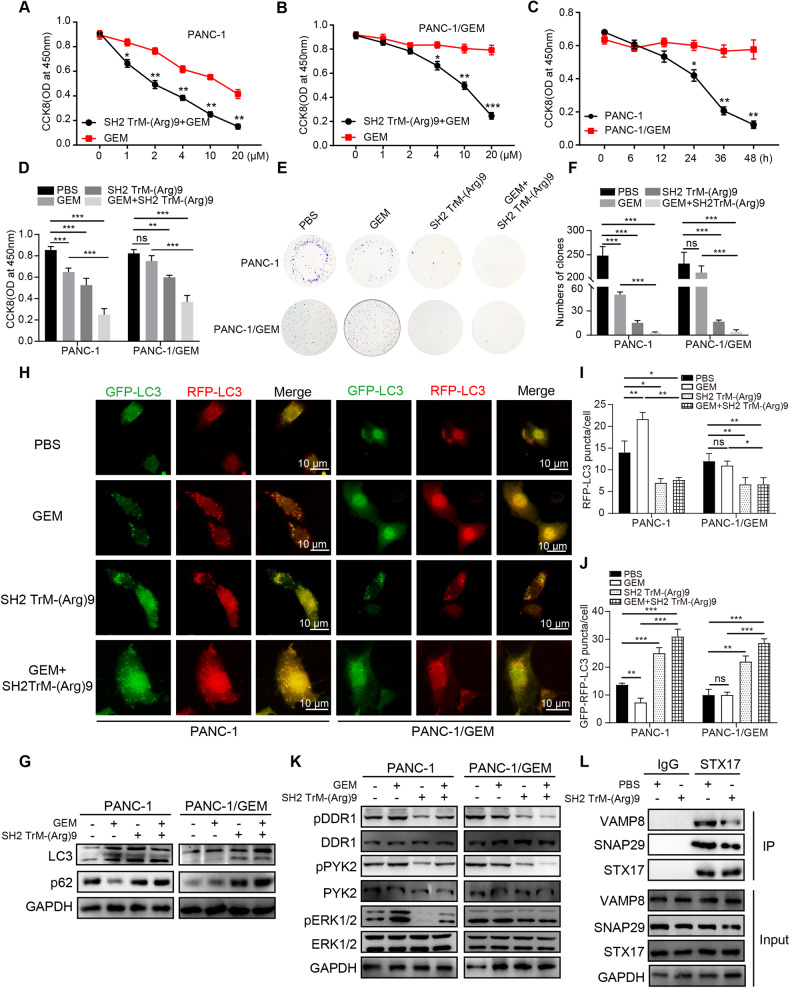
SH2 TrM-(Arg)9 mediated-autophagy inhibition sensitizes PANC-1/GEM cells to gemcitabine in vitro. **A**, **B** Changes in cell proliferation between PANC-1 (**A**) and PANC-1/GEM (**B**) (PANC-1-gemcitabine resistant) cells after incubated with gemcitabine (T0251, Targetmol, USA) at different concentrations determined by CCK-8 assay. Cells were treated with SH2 TrM-(Arg)9 (1 μM) plus gemcitabine or gemcitabine-only (GEM) for 4 h. **C** Changes in cell proliferation between PANC-1 and PANC-1/GEM cells after incubation with GEM for different periods determined by CCK-8 assay. **D** Effects of SH2 TrM-(Arg)9, GEM, and (GEM + SH2 TrM-(Arg)9) treatments on the proliferation of PANC-1 (left) and PANC-1/GEM (right) cells. Cell viability was measured by CCK-8 assays. **E** Representative images of colony formation assay showing colonies formed by PANC-1 (up) and PANC-1/GEM (down) cells incubated with SH2 TrM-(Arg)9, GEM, and GEM + SH2 TrM-(Arg)9 treatments. **F** Bar graph depicting the change in the number of cell colonies. (*n* = 3). **G** Western blot of LC3 and p62 after treated with SH2 TrM-(Arg)9, GEM, and (GEM + SH2 TrM-(Arg)9). **H**–**J** Representative fluorescence images of red-only and the yellow puncta in PANC-1 and PANC-1/GEM cells after SH2 TrM-(Arg)9, GEM, and (GEM + SH2 TrM-(Arg)9) treatment. **K** Western blot of pDDR1, DDR1, pPYK2, PYK2, pERK1/2, and ERK1/2 after treated with SH2 TrM-(Arg)9, GEM and (GEM + SH2 TrM-(Arg)9). **L** Co-immunoprecipitation of STX17 with VAMP8 and SNAP29 from PANC-1/GEM WCL upon SH2 TrM-(Arg)9 treatment. IgG as control. The gemcitabine usage condition of Fig. 5D-K were 4 μM, 24 h. The SH2 TrM-(Arg)9 condition of Fig. 5D-L were 1 μM, 2 h. Results are representative of three independent experiments with similar results. ^*^P < 0.05, ^**^P < 0.01, ^***^P < 0.001.

Gemcitabine (GEM) is effective in most pancreatic cancer patients, but gradually emerging tumor resistance to GEM severely limits its efficacy [48]. To test whether autophagy blockade sensitizes PDAC cells to GEM, cell viability was examined in PANC-1 and PANC-1/GEM cells (GEM-resistant cells). SH2 TrM-(Arg)9 (1 μM, 2 h), GEM, and their combination (GEM + SH2 TrM-(Arg)9) were administered to PANC-1 and PANC-1/GEM cells to confirm the proper use conditions of GEM (4 μM, 24 h) (Fig. 5A-C). The survival rates of (GEM + SH2 TrM-(Arg)9)-treated cells were lower than those of the control groups (PBS, GEM, and SH2 TrM-(Arg)9) (Fig. 5D). Colony formation assays showed similar results (Fig. 5E, F). The data showed SH2 TrM-(Arg)9 increased the sensitivity of PANC-1/GEM cells to GEM. Cells treated with GEM had a lower p62 level in PANC-1 cells, whereas SH2 TrM-(Arg)9 or (SH2 TrM-(Arg)9 + GEM) increased the p62 and LC3-II/LC3-I ratio (Fig. 5G). These data suggested GEM promoted autophagy, while SH2 TrM-(Arg)9 attenuated GEM-induced autophagy.

To further confirm the inhibitory effect of SH2 TrM-(Arg)9 on GEM-induced autophagy, we visualized autophagic flux using the GFP-mRFP-LC3 system [10]. The green dots were significantly increased after GEM administration in PANC-1 cells, suggesting the enhancement of autophagy. However, SH2 TrM-(Arg)9 increased the number of autophagosomes (GFP-RFP-LC3, yellow puncta) but not autolysosomes (RFP-LC3, red puncta), indicating autophagy inhibition. When cells were treated with both SH2 TrM-(Arg)9 and GEM, the number of autophagosomes increased, but autolysosomes decreased. When cells were treated with both SH2 TrM-(Arg)9 and GEM, the results reaffirmed SH2 TrM-(Arg)9 diminished GEM-induced autophagy (Fig. 5H–J).

Furthermore, pDDR1, pPYK2, and pERK1/2 were decreased by SH2 TrM-(Arg)9 even in PANC-1/GEM cells (Fig. 5K), which showed that the blocking effect of SH2 TrM-(Arg)9 in DDR1 signaling pathway did not affected by GEM. PF431396 and Selumetinib reversed the autophagy process induced by collagen I (Fig. S4A, B), suggesting that SH2 TrM-(Arg)9 inhibited autophagic flux mediated by the DDR1/PYK2/ERK signaling. Meanwhile, SH2 TrM-(Arg)9 impaired the SNAP29/STX17/VAMP8 complex formation (Fig. 5L). We also found SH2 TrM-(Arg)9 decreased P-gp (Fig. S4C). All data above implied SH2 TrM-(Arg)9 may be a novel therapy for tumor resistance through autophagy inhibition.

### Combination of SH2 TrM-(Arg)9 and GEM exhibits satisfactory anti-tumor efficacy in vivo

**Fig. 6:**
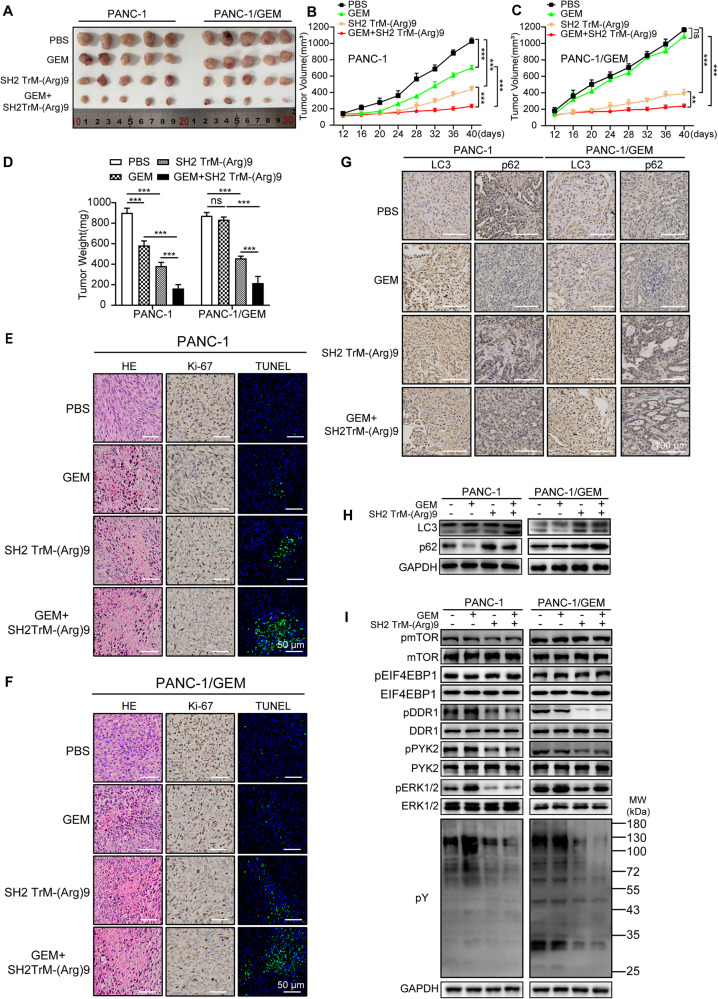
SH2 TrM-(Arg)9 plus gemcitabine exhibits satisfactory anti-tumor efficacy in vivo. **A** Excised tumors of PANC-1 and PANC-1/GEM xenografts mouse models in different groups are shown. Mice were injected with different treatments containing GEM (50 mg/kg), SH2 TrM-(Arg)9 (5 mg/kg), or SH2 TrM-(Arg)9 (5 mg/kg) +GEM (20 mg/kg). **B**, **C** The tumor volumes of PANC-1 (**B**) and PANC-1/GEM (**C**) mouse models in different groups are shown (*n* = 5). **D** Tumor weights of PANC-1 and PANC-1/GEM mice in different groups. (*n* = 5). **E**, **F** Representative histological examination of the dissected tumors using HE staining, TUNEL assay, and Ki67 staining of PANC-1 (**E**) and PANC-1/GEM (**F**) mouse models. **G** Representative histological examination of the dissected tumors using LC3 and p62 staining. **H** Western blot of LC3 and p62 in tumors with different treatments. **I** Western blot of pY, pmTOR, mTOR, pEIF4EBP1, EIF4EBP1, pDDR1, DDR1, pPYK2, PYK2, pERK1/2, and ERK1/2 in tumors with different treatments. (*n* = 5). Results are representative of three independent experiments with similar results. ^**^P < 0.01, ^***^P < 0.001.

To further confirm that the inhibition of autophagy is a potential therapeutic approach against PDAC in vivo, PANC-1 and PANC-1/GEM cells were inoculated subcutaneously into NOD/SCID mice. Compared with the tumor volume of mice inoculated with the PBS and GEM control groups, (SH2 TrM-(Arg)9 + GEM) markedly reduced the tumor volumes (Fig. 6A–D). The histological data showed (SH2 TrM-(Arg)9 + GEM) resulted in a higher level of necrotic lesions, lower level of proliferation ability, and higher apoptosis rate than the other groups (Fig. 6E, F). This result indicated SH2 TrM-(Arg)9 enhanced the anti-tumor effect of GEM. Moreover, p62 and LC3-II/LC3-I ratio increased in tumors in SH2 TrM-(Arg)9 and (SH2 TrM-(Arg)9 + GEM)- treated groups (Fig. 6G, H). mTOR signaling was also not influenced simultaneously and was consistent with the data in cells (Fig. 6I), meaning that SH2 TrM-(Arg)9 affected autophagy without conditional regulation of the mTOR signaling pathway. Treatment with (SH2 TrM-(Arg)9 + GEM) decreased pDDR1, pPYK2, and pERK1/2 of tumors in PANC-1 and PANC-1/GEM mouse models (Fig. 6I). Therefore, SH2 TrM-(Arg)9 boosted the susceptibility of PDAC cells to GEM, thereby enhancing the efficacy of GEM against pancreatic cancer.

Safety is crucial for guiding the clinical use of drugs. To evaluate the side effects of (SH2 TrM-(Arg)9 + GEM), hemanalysis was performed with blood from mice when they were sacrificed. In PANC-1 xenograft model, compared to GEM-treated mice, (SH2 TrM-(Arg)9 + GEM) decreased levels of Alanine aminotransferase (ALT) and Aspartate aminotransferase (AST) (Fig. S5A, B); and (SH2 TrM-(Arg)9 + GEM) increased levels of red and white blood cell (RBC and WBC) number, hemoglobin (HGB), platelet (PLT) number, monocyte, and other indexes (Fig. S5C–F). In contrast, GEM alone affected indicators above (Fig. S5A–F). In PANC-1/GEM xenograft mice, GEM-treated mice showed a similar performance to that of the PBS group. Among different groups, (SH2 TrM-(Arg)9 + GEM) reduced the levels of ALT and AST (Fig. S5G, H), and increased RBC, WBC, HGB, and PLT (Fig. S5I–L and Dataset 2).

## Discussion

**Fig. 7:**
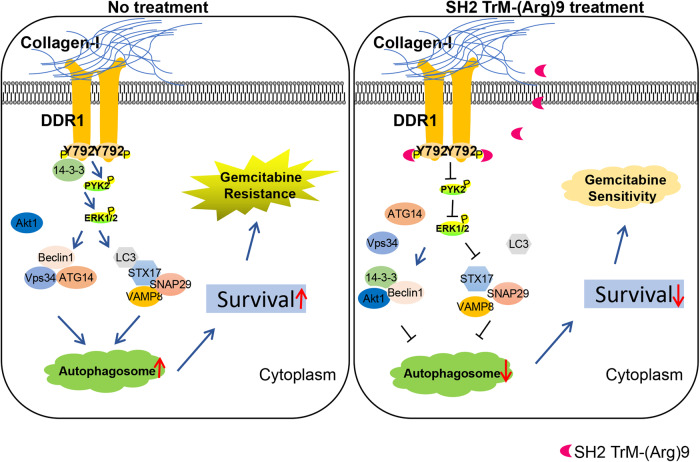
Schematic diagram of SH2 TrM-(Arg)9 inhibiting autophagy of PDAC cells. SH2 TrM-(Arg)9 mediated autophagosome-lysosome fusion inhibition via DDR1/PYK2/ERK signaling.

Autophagy is a widespread self-digestion process that has been retained in the evolution of organisms, through which cells can meet the needs of metabolism and organelle renewal [49]. In the environment of hypoxia, nutritional deficiency, metabolic stress, and anticancer treatment, cancer cells will survive by autophagy [50]. Our study confirmed activation of collagen-DDR1-mediated signaling promoted autophagic flux in pancreatic cancers and prolonged the survival of PDAC cells. We also identified DDR1, together with the 14-3-3/Beclin1/Akt1 protein complex, is required for collagen-mediated DDR1/PYK2/ERK signaling, whereas SH2 TrM-(Arg)9 inhibited autophagic flux and regulated autophagy-associated therapy sensitivity (Fig. 7). Our experiments uncovered a DDR1-driven mechanism that eliminates PDAC therapy resistance, and laid the groundwork for the development of therapy-sensitizing agents.

Compared with the SH2 domain, the SH2 superbinder has a stronger binding ability for the pY site and further regulates the activation process of the cell [17]. We pioneered the integration of SH2 superbinder with a cell penetrating peptide (CPP), to allow it to pass through cell membranes and SH2 TrM-(Arg)9 has a wide range of anticancer effects in melanoma, pancreatic cancer, non-small cell lung cancer, and other cells [17, 18, 51, 52]. The specific mechanism of action involves cell proliferation, migration, and apoptosis [17, 18]. What’s more, SH2 TrM-(Arg)9 has little toxic effect on normal cells. It is a promising broad-spectrum cancer drug. Our group has long maintained the improvement of the function and safety of SH2 superbinder [17, 18, 51, 52]. Here we focused on the relationship between SH2 superbinder and autophagy. Other researchers have achieved treatment of pancreatic cancer by inhibiting autophagy [13, 27]. In light of these previous findings, we hypothesized and was able to verify SH2 superbinder affected autophagy by regulating the DDR1/PYK2/ERK signaling. Since the SH2 superbinder used in this study did not conjugate specific aptamers [18] targeting tumor cells, its targeting ability could only rely on the EPR effect (enhanced permeability and retention effect) of the tumor.

Additionally, SH2 TrM-(Arg)9 significantly reduced the side effects of GEM and increased the sensitivity of cells to GEM in vitro and in vivo. To ensure the synergistic anti-tumor effect of GEM, a lower dose was used to reduce its toxicity and side effects (Fig. S5). Studies on conjugating aptamers have been described in our previous paper [18]. This study focuses on revealing the effect of the SH2 superbinder on autophagy, so there are no experiments on targeting.

Post-translational modifications of proteins, such as phosphorylation, acetylation, and ubiquitination, are often associated with the autophagy process, and phosphorylation mainly focuses on serine and threonine [53]. Although tyrosine phosphorylation accounts for a small proportion of protein phosphorylation, it also plays an important role in cancer development [54]. However, the relationship between autophagy and phosphorylation has been less studied [55].

In conclusion, we analyzed the mechanism of action of the SH2 superbinder in-depth. For the first time, SH2 superbinder was found to have an anticancer role by affecting autophagy in PDAC cells and animal models. When combined with GEM, SH2 superbinder reduced chemotherapy resistance and side effects. Based on the significant autophagy inhibition effect of SH2 superbinder on PDAC cells, we provided evidence to support using SH2 superbinder as an anticancer drug once again, providing a new direction for the clinical treatment of PDAC.

## Materials and methods

### Construction, expression and purification of GST fusion proteins

SH2 superbinder (SH2 TrM) has three amino acids (AAs) mutations compared with wild type SH2 domain of Src (SH2 WT). Both SH2 WT and SH2 TrM were modified with Nona-arginine (Arg)9, a cell-penetrating peptide (CPP). Genes encoding SH2 WT/TrM from the pEGFP-C3-Src SH2 WT/TrM plasmids were subcloned into the pGEX-4T3 vector to express GST-Src-SH2 WT/TrM-(Arg)9 proteins. SH2 WT/TrM-(Arg)9 were used as abbreviation. Amino acid sequences and primers related are listed in Table S1 and Table S2, and the schematic diagram of pGEX-4T3-SH2 WT/TrM-(Arg)9 is shown in Fig. S6.

All amplified products were purified using the Gel Band Purification Kit (Beijing ComWin Biotech). The backbone was digested with BamHI (Cat. #R3136V) and HindIII (Cat. #R3104V) at 37 °C for 2 h, and the purified PCR products were reacted with the linearized pGEX-4T3 vector at 37 °C for 30 min. The experiment used the One Step Cloning Kit (Vazyme, Nanjing), according to the principle of homologous recombination.

The specific procedure of plasmid transformation, protein expression and purification can refer to the previous study [17]. The BCA Protein Assay Kit was used to quantify the protein concentration in cell homogenates. Immediately prior to use in biological assays, protein purity was verified by SDS-PAGE using Coomassie brilliant blue staining intensity.

### Transmission electron microscopy

Cells from different groups were fixed with 2.5% glutaraldehyde solution according to the previous description [56]. Images were captured by a transmission electron microscope (TEM, JEOL, Tokyo, Japan).

### In vitro DDR1 kinase assay

The specific DDR1 kinase activity in the presence and absence of SH2 TrM-(Arg)9 was measured using a Universal Kinase Activity Kit (R&D Systems, Cat. #EA004). The assay measures the inorganic phosphate that is released from ADP via substrate phosphorylation by DDR1. The specific procedures are carried out according to the instructions.

### Patients and sample collection

PDAC and the adjacent tissues were obtained from patients who underwent surgical resection for PDAC at the Tongji Hospital (Wuhan, China). Tissue acquisition and handling of human tissue specimens used in this study have been approved by the Ethics Committee of Tongji Medical College, Huazhong University of Science and Technology. Patient information on clinical specimens is listed in Table S3.

### Statistics

Results are presented as mean ± standard deviation (SD) and analyzed. The student’s *t* test was used for statistical significance of the differences between the different groups. *P* values of less than 0.05 were considered statistically significant. *P < 0.05, **P < 0.01 and ***P < 0.001.

Other methods are provided in Supplementary File 1.

### Reporting summary

Further information on research design is available in the Nature Research Reporting Summary linked to this article.

### Supplementary information

SUPPLEMENTAL FILE 1

SUPPLEMENTAL FILE 2

SUPPLEMENTAL FILE 3

Original western blot

Reporting Summary

## Data Availability

Data were generated by the authors and available on request.
