# Supplementary material for: Blockade of DDR1/PYK2/ERK signaling suggesting SH2 superbinder as a novel autophagy inhibitor for pancreatic cancer
Source: Cell Death Dis. 2023 Dec 9;14(12):811. doi: 10.1038/s41419-023-06344-4 (PMC10710504; doi:10.1038/s41419-023-06344-4)
Supplement: Supplementary file 1 — SUPPLEMENTAL FILE 1 [file 41419_2023_6344_MOESM1_ESM.pdf]

Blockade of DDR1/PYK2/ERK signaling suggesting SH2 superbinder as a novel autophagy inhibitor for pancreatic cancer

Hui Xu[1,2#], Ming Tan[2#], Guo-Qing Hou[1], Ya-Zhou Sang[1], Li Lin[1], Xiao-Cai Gan[1], Xuan Cao[2,3*], An-Dong Liu [1,4*]

Supplementary Methods

Cell culture and administration

BxPC-3, PANC-1, ASPC-1 and hTERT-HPNE were obtained from American Type Culture Collection (ATCC). The human pancreatic duct epithelial (HPDE) cells were obtained from patients with PDAC who underwent surgical resection at Wuhan Union Hospital. The culture condition for the hTERT-HPNE cells and the primary HPDE cells was followed the procedures previously reported[1]. The full culture medium of MCF10A cell line contained DMEM:F12 medium was supplemented with antibiotics, EGF (10 ng/mL), insulin (10 µg/ mL), cholera toxin (1 µg/ mL), hydrocortisone (1 µg/ mL) and heat-inactivated horse serum (5%; Invitrogen). Gemcitabine-resistant PANC-1 (PANC-1/GEM) cells were established by our laboratory as previously reported[2]. Briefly, PANC-1 cells were exposed to 0.2 µM of gemcitabine for one week. When cells returned to a normal growth rate, the concentration of gemcitabine (T0251, Targetmol, USA) was gradually increased to 5, 10, and 20 µM until cells became resistant to 20 µM of gemcitabine for six months. Cells were cultured in DMEM medium supplemented with 10% fetal bovine serum and 100 U/mL

penicillin-streptomycin. All cell lines were cultured under 5% CO_2 at 37°C.

Plasmids construction, siRNAs and transfection

DNA fragments encoding DDR1 and DDR1(Y792F) were synthesized by the Beijing Genomics Institute (Beijing, China), and sub-cloned into the pCMV-Flag plasmid. DNA fragments encoding STX17 and LC3 were amplified by PCR from the genomic DNA of PANC-1 cells, and sub-cloned into the pcDNA3.1-HA (STX17), pEGFP-C3 (LC3), and pmCherry-C1-EGFP (LC3) plasmids, respectively. The nucleotide sequences of all constructs were confirmed by DNA sequencing. The oligonucleotides targeting DDR1 mRNA (sense:5'-GUAUUUAUCUGAGGCCGUGUATT-3', antisense:5'-UACACGGCCUCAGAUAAAUACTT-3') and negative control (sense:5'-UUCUCCGAACGUGUCACGUTT-3', antisense:5'-ACGUGACACGUUCGGAGAATT-3') were synthesized by Sangon Biotech (Shanghai, China), and transfected into cells with Lipofectamine RNAiMAX reagent (Invitrogen) according to the manufacturer's protocol. Lipofectamine 2000 Transfection Reagent (Invitrogen) was used to transfect the cells with the plasmids according to the manufacturer's protocol.

Western blot and Immunoprecipitation

Cells were lysed on ice using lysis buffer (1% NP-40, 50 mM Tris-HCl (pH 7.4), 150 mM NaCl, 2 mM EDTA, 50 mM NaF, 10% glycerol, and the complete protease inhibitor cocktail); then the lysate proteins were collected and further analyzed. Protein A/G agarose beads were used for immunoprecipitation analysis. Western blot was performed according to standard method with primary antibodies against pY

(Abcam EPR16871), pmTOR (CST#5536), mTOR (66888-1-Ig, Proteintech), pEIF4EBP1 (CST#2855), EIF4EBP1 (Proteintech, 60246-1-Ig), ATG14 (Proteintech, 28021-1-AP), 14-3-3 (Proteintech, 66061-1-AP), Akt1 (CST#2938), pSTAT3 (CST#4113), STAT3 (CST#9139), pJAK2 (CST#3771), JAK2 (CST#3230), pDDR1(Y792) (ABclonal#AP1072), pDDR1(Y513) (ABclonal#AP1265), DDR1(CST#5583), pPYK2 (ABclonal#AP0612), PYK2 (17592-1-AP, Proteintech), pSrc (CST#6943), Src (CST#2110), STX17 (17815-1-AP, Proteintech), SNAP29 (12704-1-AP, Proteintech), VAMP8 (15546-1-AP, Proteintech), LC3 (CST#12741), p62 (CST#23214), Beclin1 (CST#4122), Beclin1(66665-1-Ig, Proteintech), Vps34 (CST#4263), P-gp (22336-1-AP, Proteintech), NRF2 (16396-1-AP, Proteintech), KEAP1 (60027-1-AP, Proteintech), Bax (50599-2-Ig, Proteintech) and Bcl2 (60178-1-Ig, Proteintech), pERK1/2 (CST#4370), ERK1/2 (CST#4695), HA (66006-2-Ig, Proteintech) and GAPDH (CST#5174) were used at recommended dilutions and then incubated with the membranes overnight at 4°C. Membranes were washed three times for 10 min each and incubated with a 1:5000 dilution of HRP-conjugated anti-mouse or anti-rabbit antibodies. Blots were washed three times with 1×TBST and analyzed using the ECL system.

Immunofluorescence

1×10^4 cells were seeded in a 12-well plate and cultured for 24 h. First, cells were incubated with different treatments. After washing with cold washing buffer, cells were first fixed in 4% formaldehyde at room temperature for 1 h, and then were permeabilized with 0.5% Triton X-100 for 30 min. Subsequently, primary and

secondary antibodies were used to stain cells. Finally, after incubated with rhodamine phalloidin (Invitrogen, 1:50) for 30 min, samples were stained with DAPI for 5 min and visualized with a fluorescence microscope (Olympus, Japan). The images were analyzed with the ImageJ software.

CCK-8 assay

Cells collected in the logarithmic phase were plated into 96-well plates (5×10^3 cells/well). On the following day, add different treatments into the cell culture medium. After incubating for various periods, 10 μL CCK-8 solution (Dojindo) was added to each well and incubated for 1~4 h. The optical absorbance was measured at the wavelength of 450 nm.

Colony formation assay

Cells were trypsinized and plated in 6-well plates (100 cells/well), incubated with different agents, and counted 14 days after seeding. The colonies were subsequently fixed with 4% formaldehyde and stained with 0.01% crystal violet for 10 min.

EdU assay

Cells were seeded in 24-well plates and then incubated with 10 μM EdU for 2 h. Next, the cells were fixed with 4% formaldehyde at 37℃ for 20 min, followed by permeabilization in 0.5% Triton X-100 for 15 min. Then 100 μL of Click-iT was added and incubated for 30 min in the dark at room temperature. After washing with PBS, the nuclei were stained with DAPI for 5 min, and images were captured by fluorescence microscope (Olympus, Japan). All procedures followed the manufacturer's instructions for the EdU kit (Abbkine Scientific, Wuhan, China).

Apoptosis analysis

Cells were sedimented by centrifugation, resuspended, and fixed in 100 μL binding buffer. Cell density in the cell suspension was adjusted to 2×10^3 cells/μL. Subsequently, 5 μL Annexin V-FITC (Fluorescein Isothiocyanate) was added to the cell suspension, followed by gentle vortexing and incubation for 10 min at room temperature in the dark. The cell suspension was then incubated with 5 μL Propidiumiodide (PI). Cells were analyzed using a FACS flow cytometer (FACS BD Biosciences, Germany) for Annexin V-FITC and PI binding. Dot plots and histograms were analyzed with FlowJo software.

TdT-UTP nick end labeling (TUNEL) assays were performed with a one-step TUNEL apoptosis assay kit (Beyotime Institute of Biotechnology), according to the manufacturer's instructions. The FITC-labeled TUNEL-positive cells were imaged under a fluorescent microscope (Olympus, Japan). The cells with green fluorescence were defined as apoptotic cells. Images were analyzed with ImageJ software.

Protein Digestion for a Label-Free Experiment

For label-free experiments, proteins were extracted from the cells with or without the SH2 superbinder treatment in triplicate. Cell lysates were lysed in UA buffer. For total phosphoproteome analysis, phosphopeptides were enriched by TiO_2 microbeads. P-Tyr peptides were immunoprecipitated from phosphopeptides using anti-P-Tyr antibodies. LC-MS/MS analysis was performed on a Q Exactive mass spectrometer (Thermo Scientific) that was coupled to Easy nLC (Thermo Fisher Scientific) for 60/120/240 min, which was operated in a positive ion mode. MS data were acquired

using a data-dependent top10 method dynamically choosing the most abundant precursor ions from the survey scan (300–1800 m/z) for HCD fragmentation. The technical services were provided by APT Company (Shanghai, China).

Data analysis and presentation

MS datasets of PANC-1 cells treated with or without SH2 TrM-(Arg)9 were reanalyzed for tyrosine phosphorylation levels using TB tools software. Hierarchical clustering was performed in Persues using Euclidian distance and average linkage clustering.

Animal studies

All animal experiments were approved by the Institutional Animal Care and Use Committee of Tongji Medical College, Huazhong University of Science and Technology. Cells (1×10^6 cells for each mouse) were inoculated into nude mice. When the volume of tumors reached about 100 mm^3, all mice (NOD/SCID, 6-week-old) were randomized into different groups (n=5). Different agents (PBS, gemcitabine, or SH2 TrM-(Arg)9) were injected into the tail vein according to the experiment's requirements. $V=1/2\times W^2\times L$ (V is the volume, L is the length, and W is the width). All animals were sacrificed when the tumor size reached about 1000 mm^3.

Hematology analysis and blood biochemical assay

ALT and AST levels were assayed in serum according to the manufacturer's instructions (Nanjing Jiancheng Corp.). Routine blood tests were performed at the Servicebio Company, Wuhan, China.

IHC assay and H&E staining

These assays were conducted as described in a previous study[1]. Tumor sections were stained with specific antibodies for IHC assays. Images were captured using a microscope (Mshot, Guangzhou, China).

References

1. Liu AD, Zhou J, Bi XY, Hou GQ, Li SS, Chen Q, Xu H, Cao X: Aptamer-SH2 superbinder-based targeted therapy for pancreatic ductal adenocarcinoma. Clin Transl Med 2021, 11(3):e337.

2. Zhou C, Yi C, Yi Y, Qin W, Yan Y, Dong X, Zhang X, Huang Y, Zhang R, Wei J et al: LncRNA PVT1 promotes gemcitabine resistance of pancreatic cancer via activating Wnt/beta-catenin and autophagy pathway through modulating the miR-619-5p/Pygo2 and miR-619-5p/ATG14 axes. Mol Cancer 2020, 19(1):118.

Supplemental Figures and Legends

Figure S1.

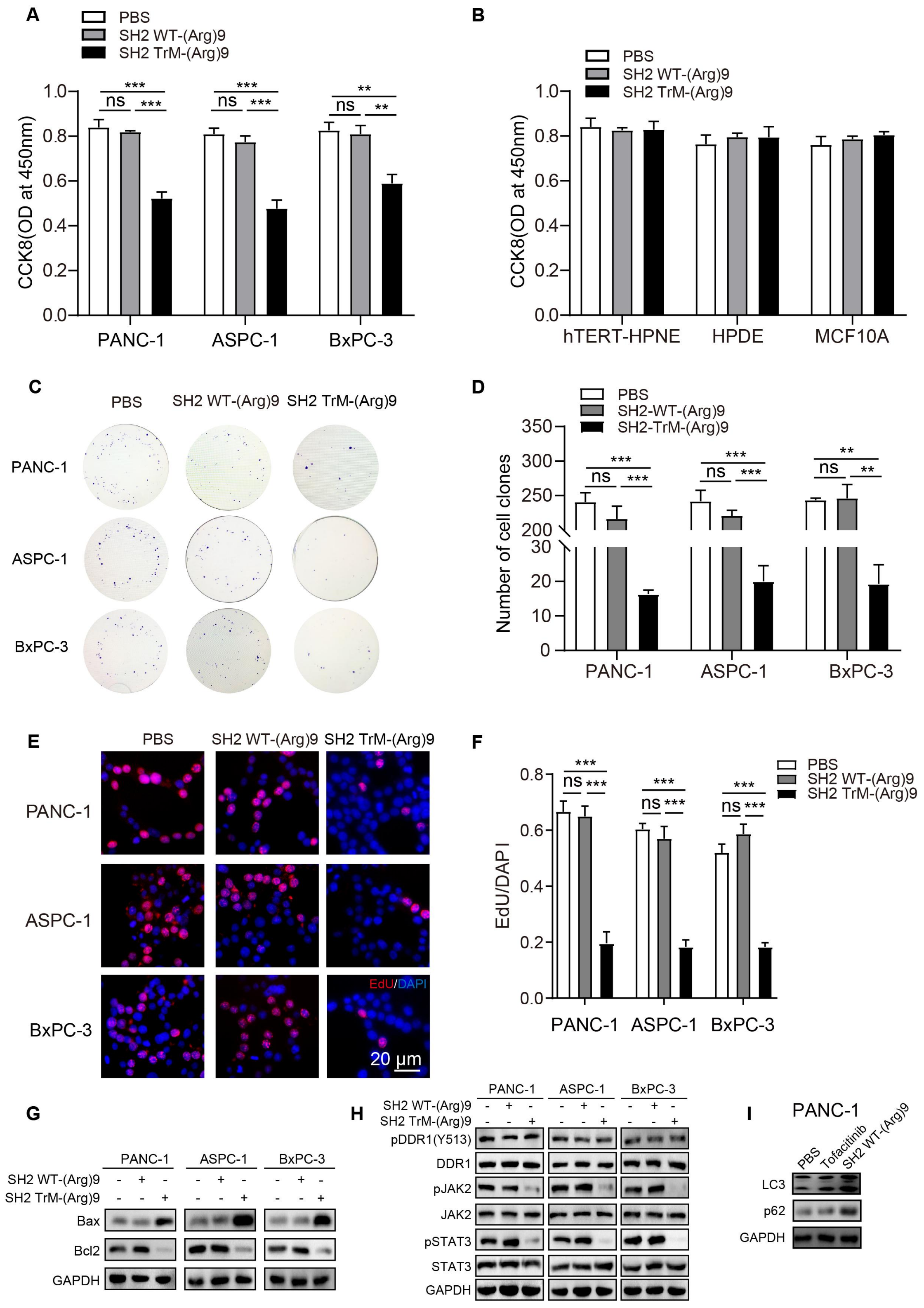

Figure S1. SH2 TrM-(Arg)9 inhibited proliferation, apoptosis and pY-mediated

signaling pathways activation.

(A-B) Cell viability was measured by CCK-8 assays. (C) Representative images of colony formation assay showing colonies formed by cells incubated with SH2 TrM-(Arg)9 and SH2 WT-(Arg)9. (D) Bar graph depicting the change in the number of cell colonies (n = 3). (E-F) Changes in cell proliferation between PDAC cells after SH2 TrM-(Arg)9 and SH2 WT-(Arg)9 incubation were determined by EdU assay (n=3). (G) Western blot of Bax and Bcl2 in PDAC cells after SH2 TrM-(Arg)9 and SH2 WT-(Arg)9 incubation. (H) Western blot of pDDR1(Y513), DDR1, pJAK2, JAK2, pSTAT3 and STAT3 in PDAC cells after SH2 TrM-(Arg)9 and SH2 WT-(Arg)9 treatment. (I) Western blot of LC3, p62 in PDAC cells after SH2 TrM-(Arg)9 and Tofacitinib treatment.The data shown are representative of three independent experiments. $^{**}P<0.01$, $^{***}P<0.001$.

Figure S2.

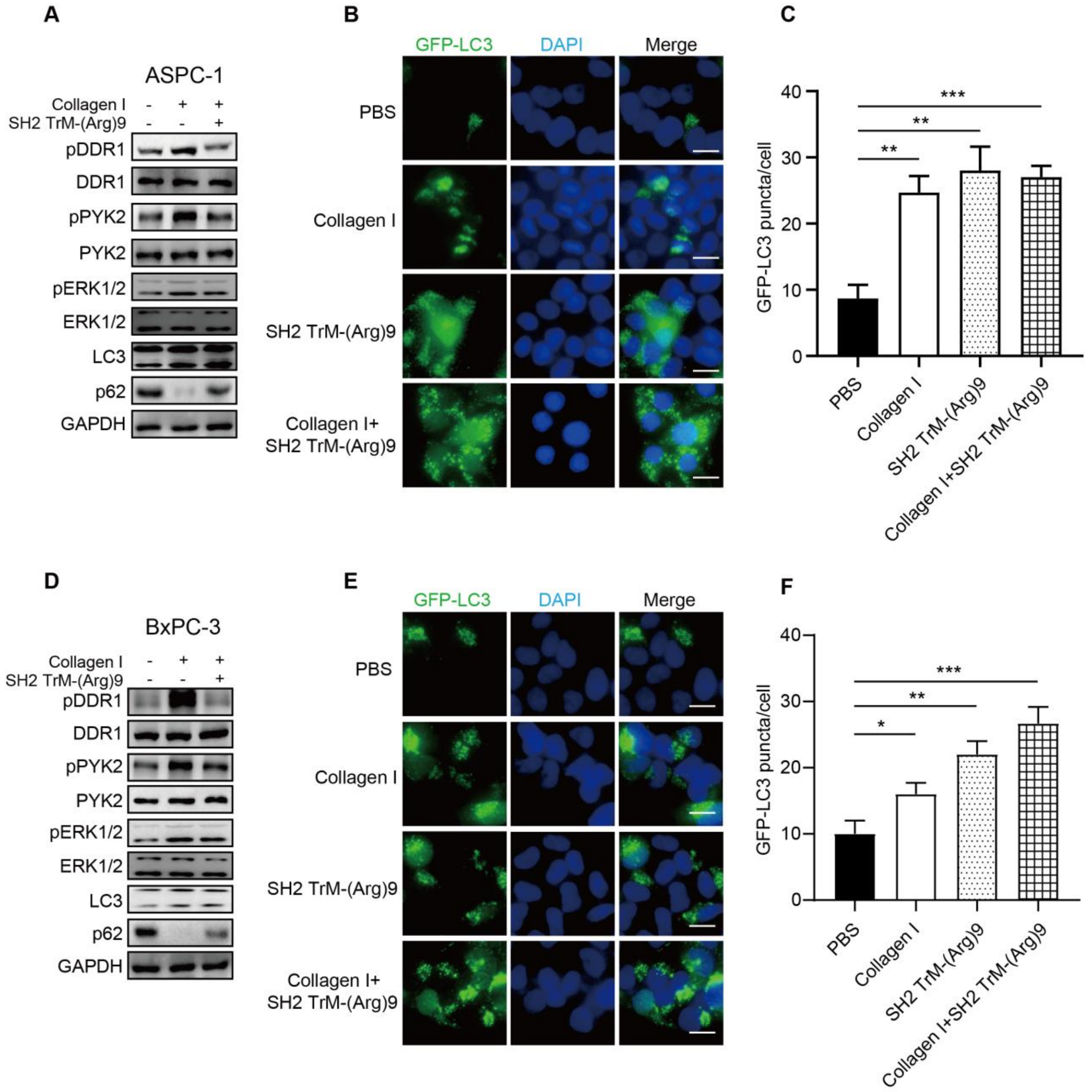

Figure S2. SH2 TrM-(Arg)9 inhibited autophagy in PDAC cells via DDR1/PYK2/ERK signaling.

(A) Western blot of pDDR1, DDR1, pPYK2, PYK2, pERK1/2, ERK1/2, LC3, and p62 after ASPC-1 cells were treated with collagen or SH2 TrM-(Arg)9. (B-C) Representative fluorescence images of GFP-LC3 in ASPC-1 cells after the treatment of collagen or SH2 TrM-(Arg)9. The number of LC3 puncta was quantified (n =3). (D) Western blot of pDDR1, DDR1, pPYK2, PYK2, pERK1/2, ERK1/2, LC3, and p62 after BxPC-3 cells were treated with collagen or SH2 TrM-(Arg)9. (E-F)

Representative fluorescence images of GFP-LC3 in BxPC-3 cells after the treatment of collagen or SH2 TrM-(Arg)9. The number of LC3 puncta was quantified (n =3). *P<0.05, **P<0.01, ***P<0.001.

Figure S3.

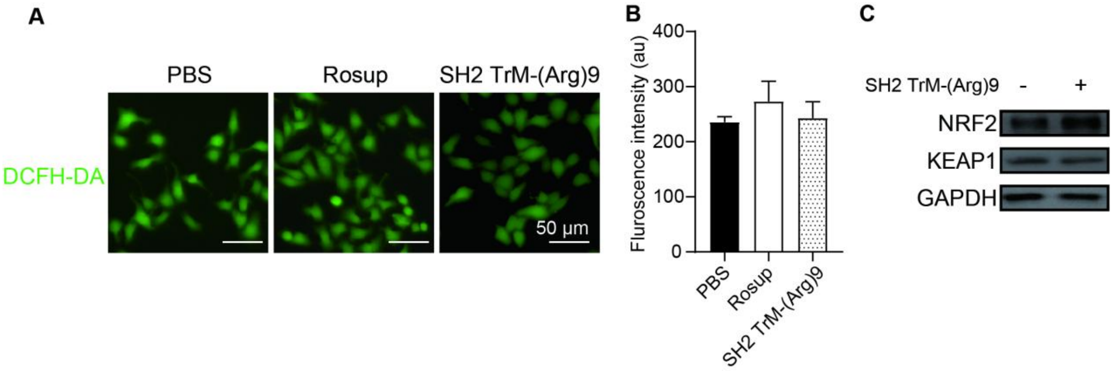

Figure S3. SH2 TrM-(Arg)9 inhibited autophagy without affecting ROS levels in PANC-1 cells.

(A) The photomicrograph of SH2 TrM-(Arg)9 mediated ROS generation indicated by increased green fluorescence in PANC-1 cells. **(B)** The graph represents the quantitative analysis of SH2 TrM-(Arg)9 induced ROS. **(C)** Western blot of NRF2 and KEAP1 after PANC-1 cells were treated with SH2 TrM-(Arg)9. The results are representative of three independent experiments with similar results.

Figure S4.

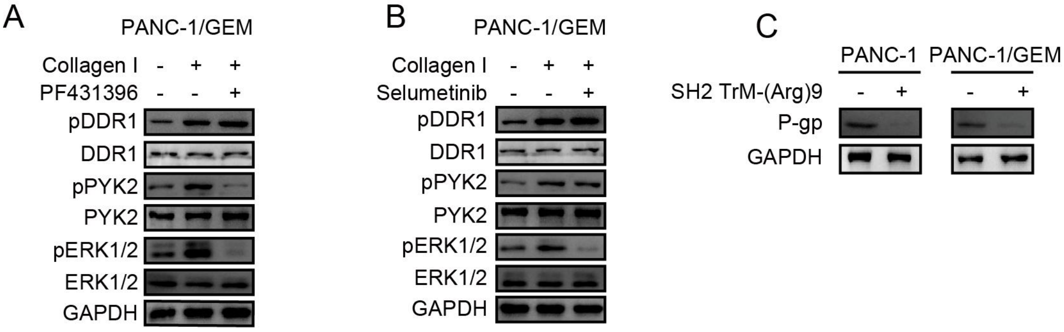

Figure S4. SH2 TrM-(Arg)9 mediated inhibition of autophagy through affecting DDR1/PYK2/ERK signaling.

(A-**B)** Western blot of pDDR1, DDR1, pPYK2, PYK2, pERK1/2, and ERK1/2 after

cells were treated with collagen or inhibitor of PYK2 (PF431396) and ERK1/2 (Selumetinib). **(C)** Western blot of P-gp after cells were treated with SH2 TrM-(Arg)9. The results are representative of three independent experiments with similar results.

Figure S5.

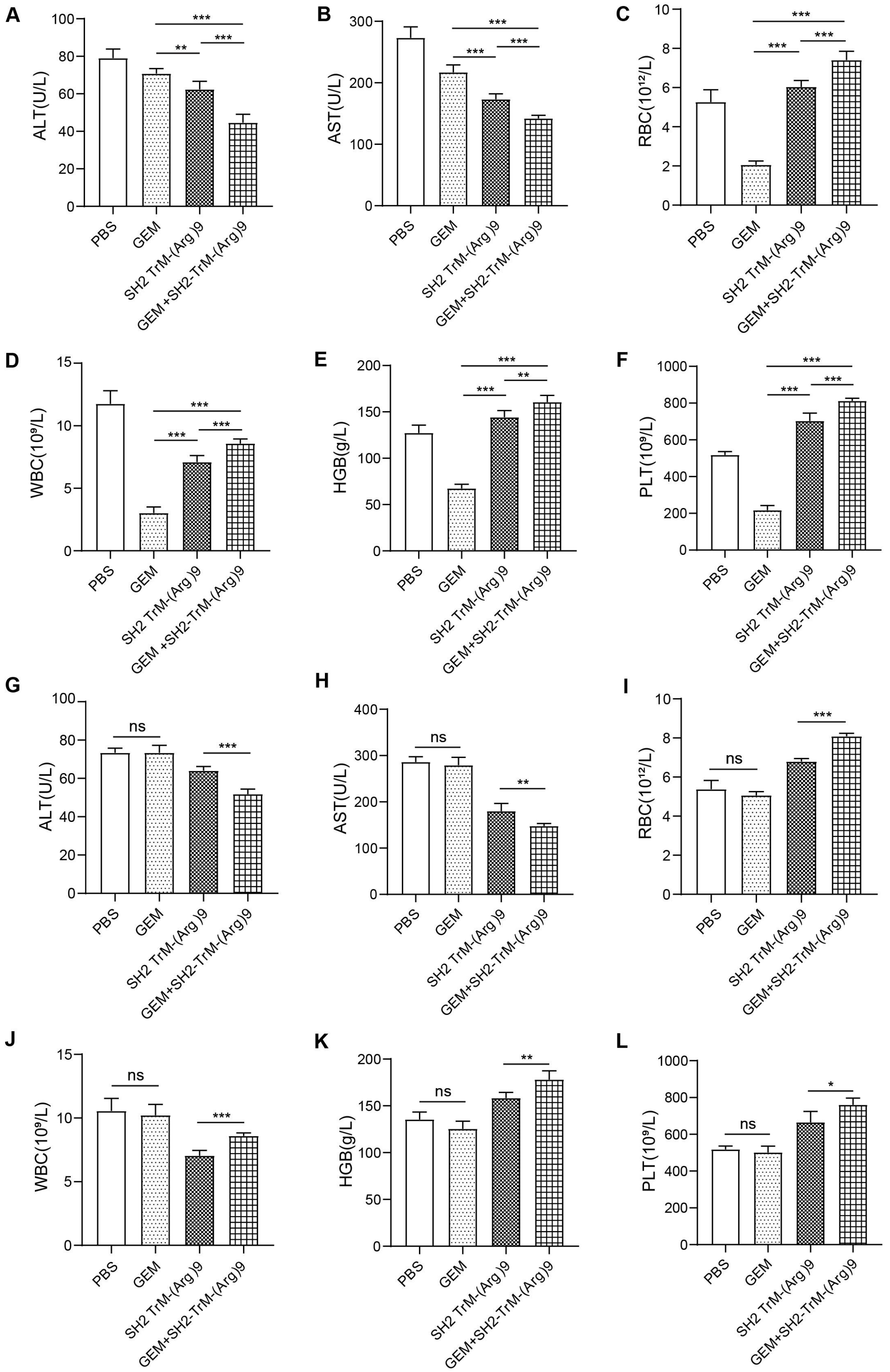
A
ALT(U/L)
B
AST(U/L)
C
RBC(10¹²/L)
D
WBC(10⁹/L)
E
HGB(g/L)
F
PLT(10⁹/L)
G
ALT(U/L)
H
AST(U/L)
I
RBC(10¹²/L)
J
WBC(10⁹/L)
K
HGB(g/L)
L
PLT(10⁹/L)
PBS
GEM
SH2 TrM-(Arg)9
GEM+SH2-TrM-(Arg)9
ns
*
**
***

Figure S5. SH2 TrM-(Arg)9 combined with gemcitabine showed slight side effects to tumor mouse model.

Hemanalysis was performed on mice when they were sacrificed. **(A-F)** Hemanalysis of PANC-1 xenograft mice. **(G-L)** Hemanalysis of PANC-1/GEM xenograft mice. *P<0.05, **P<0.01, ***P<0.001. Results are representative of three independent experiments with similar results.

Figure S6.

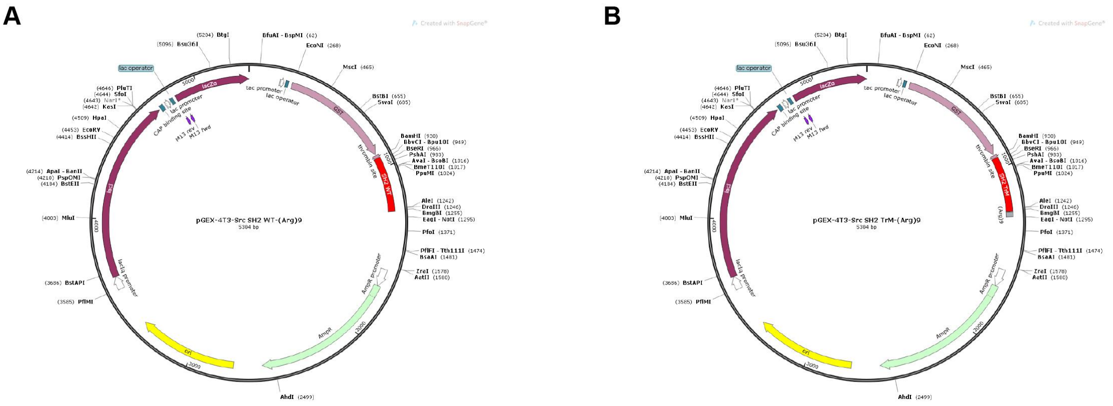

Figure S6. Schematic diagram of pGEX-4T3-SH2 WT-(Arg)9 and pGEX-4T3-SH2 TrM-(Arg)9.

(A) Schematic diagram of pGEX-4T3-SH2 WT-(Arg)9. (B) Schematic diagram of pGEX-4T3-SH2 TrM-(Arg)9

Supplemental Tables.

Table S1. Amino acid sequences of Src SH2 domain and related variants. The sequence of Src SH2 WT/TrM is underlined with a wavy line. Tripe mutant sites are marked in red and bold. The sequence of (Arg)9 is marked in green and bold. GST sequences are underlined with a straight line.

Table S1. The amino acid sequences of SH2 WT/TrM-(Arg)9 (N-C).

GST-Src SH2 WT-(Arg)9	MSPILGYWKIKGLVQPTRLLLEYLEEKYEEHLYERDEGDKWRNKKFELGLEFPNLPYYIDGDVKLTQSMAIIRYIADKHNMLGGCPKERAEISMLEGAVLDIRYGVSRIAYSKDFETLKVDFLSKLPEMLKMFEDRLCHKTYLNGDHVTHPDFMLYDALDVVLYMDPMCLDAFPKLVCFKKRIEAIPQIDKYLKSSKYIAWPLQGWQATFGGGDHPPKSDLVPRGSDSIQAEEWYFGKITRRESERLLLNAENPRGTFLVRESET**T**KGAY**C**LSVSDFDNAKGLNVKHY**K**IRKLDSGGFYITSRTQFNSLQQLVAYYSKHADGLCHRLTTVCPTSKG*RRRRRRRRR*
GST-Src SH2 TrM-(Arg)9	MSPILGYWKIKGLVQPTRLLLEYLEEKYEEHLYERDEGDKWRNKKFELGLEFPNLPYYIDGDVKLTQSMAIIRYIADKHNMLGGCPKERAEISMLEGAVLDIRYGVSRIAYSKDFETLKVDFLSKLPEMLKMFEDRLCHKTYLNGDHVTHPDFMLYDALDVVLYMDPMCLDAFPKLVCFKKRIEAIPQIDKYLKSSKYIAWPLQGWQATFGGGDHPPKSDLVPRGSDSIQAEEWYFGKITRRESERLLLNAENPRGTFLVRESET**V**KGAY**A**LSVSDFDNAKGLNVKHY**L**IRKLDSGGFYITSRTQFNSLQQLVAYYSKHADGLCHRLTTVCPTSKG*RRRRRRRRR*

The sequence of Src SH2 WT/TrM is underlined with wavy line. Tripe mutant sites are marked in **bold**. GST sequences are underlined with straight line. Sequence of (Arg)9 is represented in italics. GST-Src-SH2-WT/TrM-(Arg)9 is termed as SH2-WT/TrM-(Arg)9 in the manuscript.

Table S2. Primer sequences for recombinant plasmids. (Arg)9 was subcloned into pGEX-4T3-Src SH2 WT/TrM using the One Step Cloning Kit.

Sequences of Primers

Plasmid	Forward Primers(5'-3')	Reverse Primers(5'-3')
pGEX-4T3-Src SH2 WT-(Arg)9	ATCTGGTTCCGCGTGGATCCGACTCCATCCAGGCTGAGG	AGTCACGATGCGGCCGCTACGGCGGCGACGACGGCGGCGACGACGGCCCTTGGACGTGGGGCACAC
pGEX-4T3-Src SH2 TrM-(Arg)9	ATCTGGTTCCGCGTGGATCCGACTCCATCCAGGCTGAGG	AGTCACGATGCGGCCGCTACGGCGGCGACGACGGCGGCGACGACGGCCCTTGGACGTGGGGCACAC

Table S3. Patient information of clinical specimens.

Patient Num.	Sex	Age	Tumor Location	Classification
67664	Female	56	Pancreatic Tail	Moderately-poorly differentiated PDAC with 1/3 metastatic/examined peripancreatic lymph nodes
68269	Male	60	Pancreatic Tail	Moderately-poorly differentiated PDAC with 2/2 metastatic/examined peripancreatic lymph nodes
68099	Female	59	Pancreatic Body	Well-moderately differentiated PDAC with 1/3 metastatic/examined peripancreatic lymph nodes
67512	Female	58	Pancreatic Body	Well-moderately differentiated PDAC with 1/2 metastatic/examined peripancreatic lymph nodes and 1/13 metastatic/examined perigastric lymph nodes

PDAC, Pancreatic ductal adenocarcinoma

Dateset 1. MS datasets of phosphotyrosine in PANC-1 cells with or without SH2 TrM-(Arg)9 treatment.

Dateset 2. Hemanalysis was performed on mice when they were sacrificed.
