# Supplementary material for: Blockade of DDR1/PYK2/ERK signaling suggesting SH2 superbinder as a novel autophagy inhibitor for pancreatic cancer
Source: Cell Death Dis. 2023 Dec 9;14(12):811. doi: 10.1038/s41419-023-06344-4 (PMC10710504; doi:10.1038/s41419-023-06344-4)
Supplement: Supplementary file 4 — Original western blot [file 41419_2023_6344_MOESM4_ESM.docx]

**Fig1**

1. GST


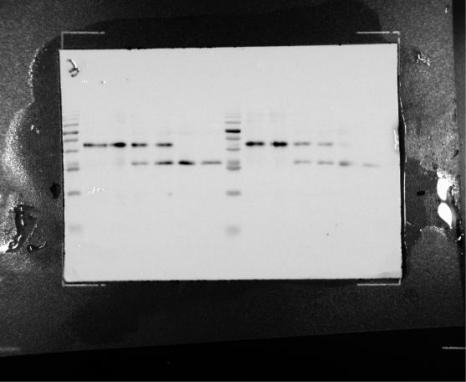


K.

Panc1

Bax


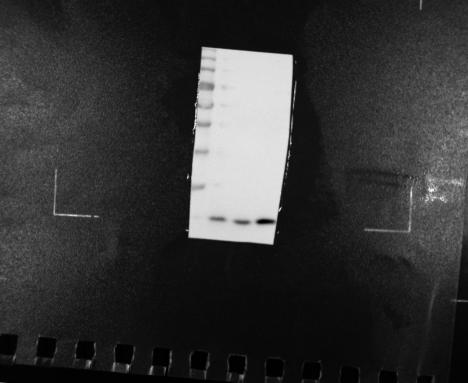


Bcl2


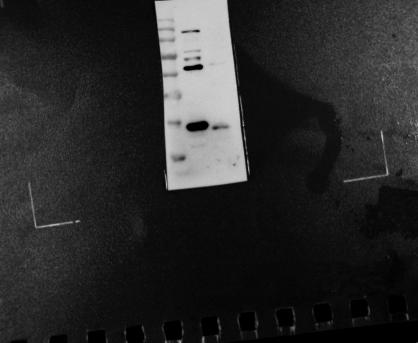


GAPDH


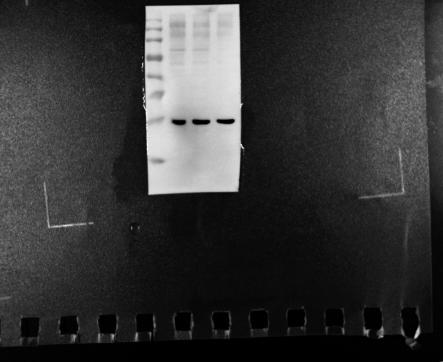


Aspc1

Bax


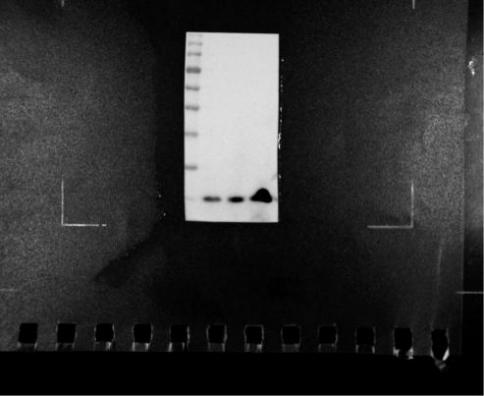


Bcl2


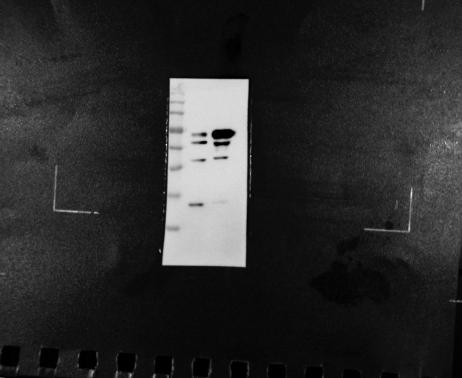


GAPDH


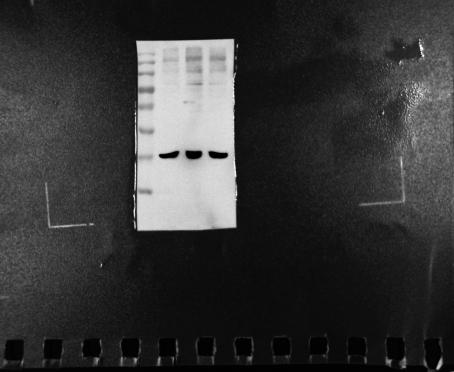


Bxpc3

Bax


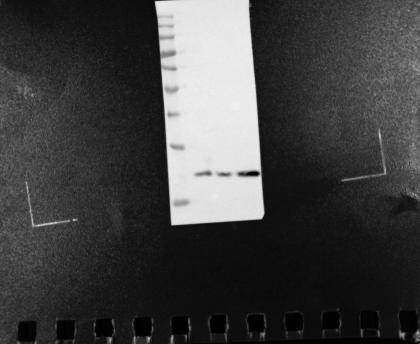


Bcl2


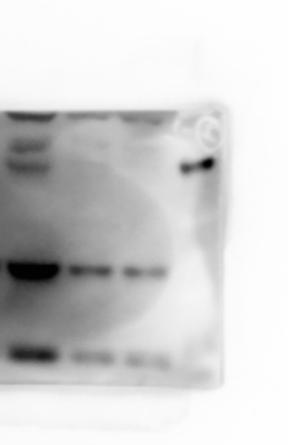


GAPDH


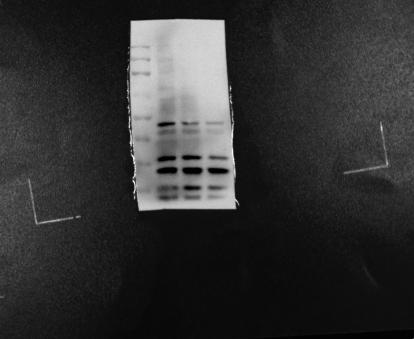


L.

Panc1

Pddr1(y513)


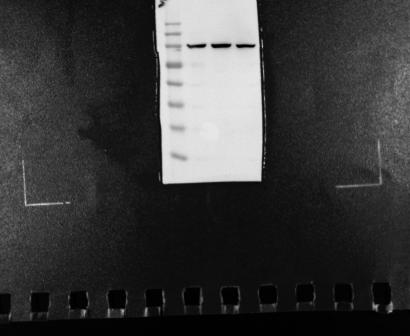


DDR1


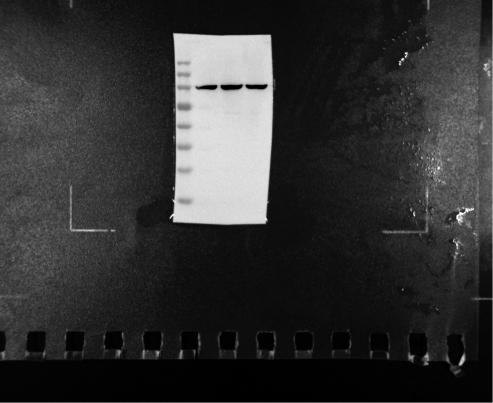


PJAK2


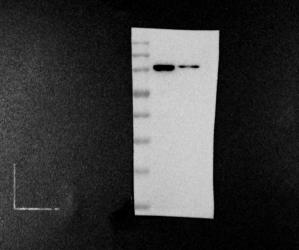


JAK2


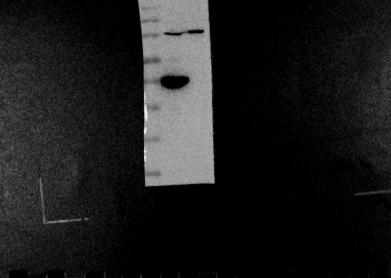


Pstat3


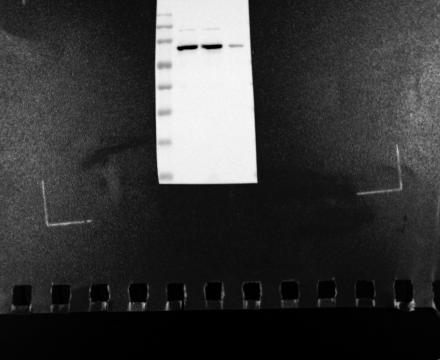


stat3


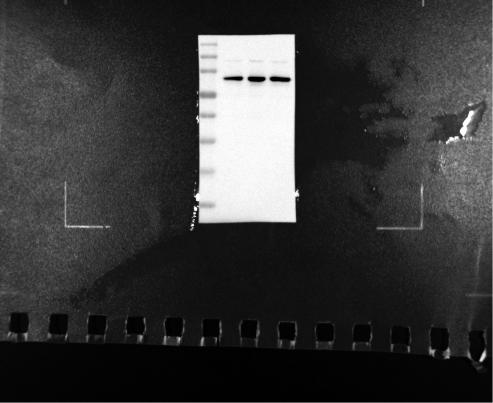


Gapdh


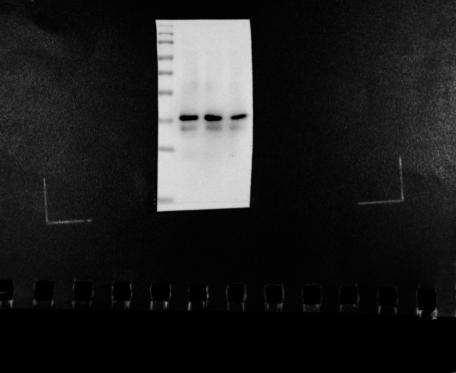


Aspc1

Pddr1(y513)


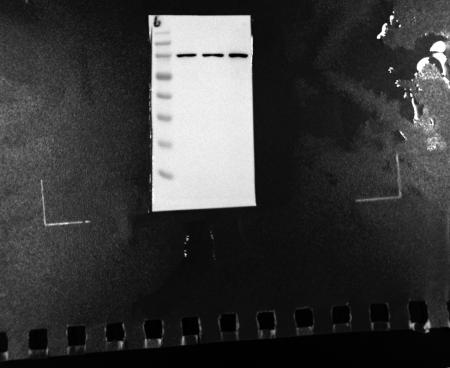


DDR1


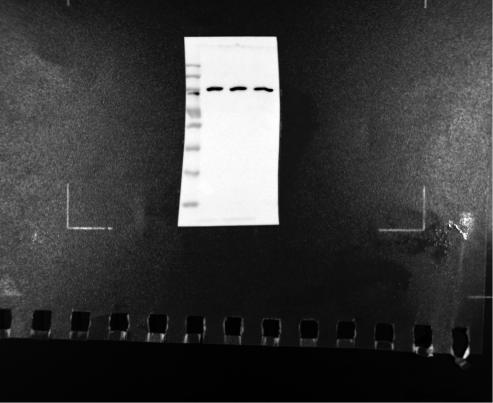


PJAK2


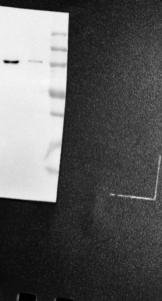


JAK2


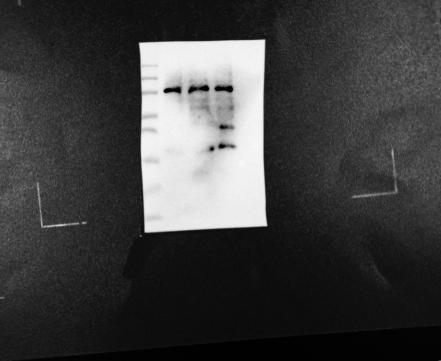


Pstat3


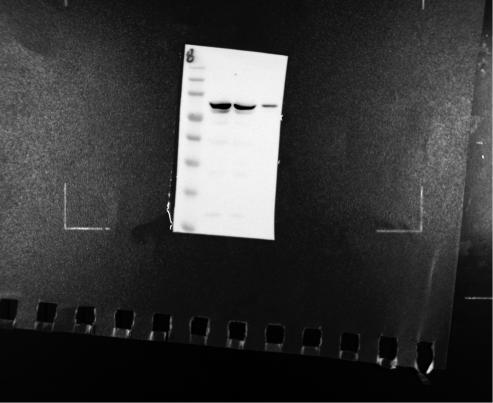


stat3


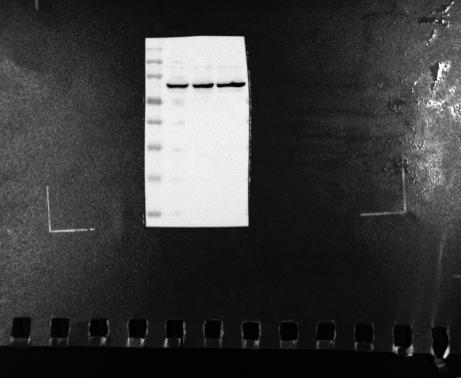


Gapdh


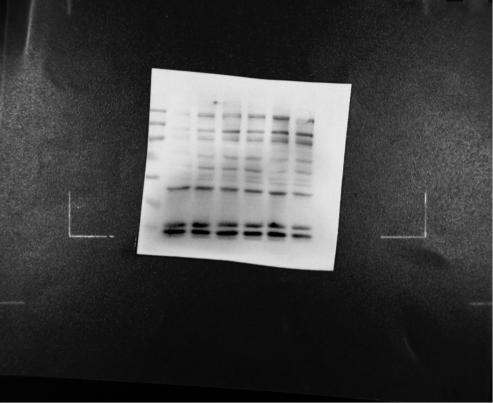


Bxpc3

Pddr1(y513)


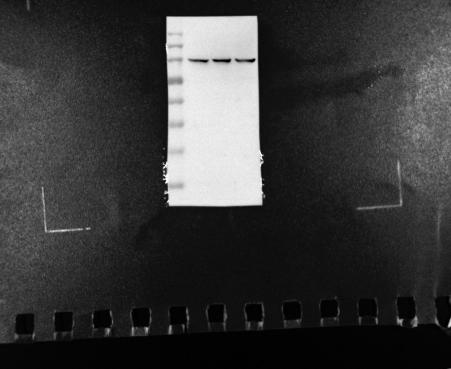


DDR1


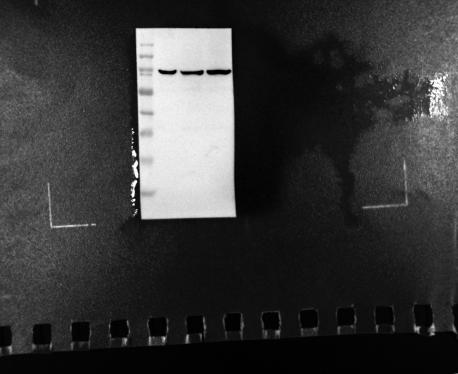


PJAK2


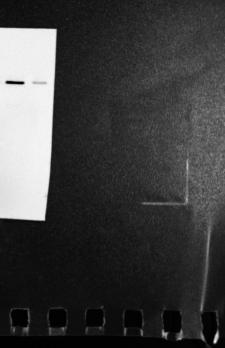


JAK2


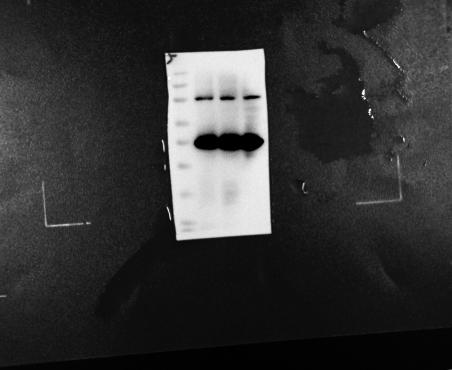


Pstat3


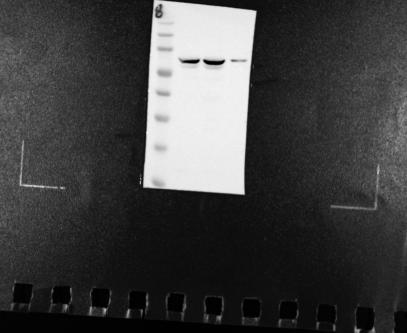


stat3


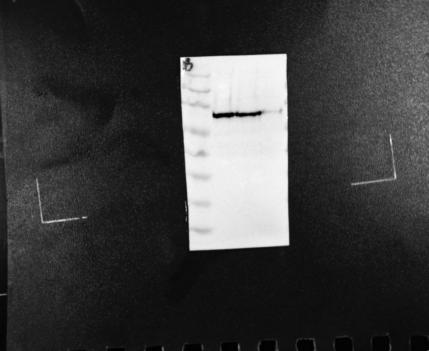


Gapdh


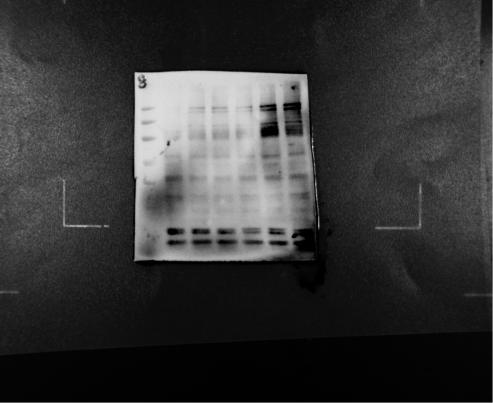


**Fig2**

C.

LC3


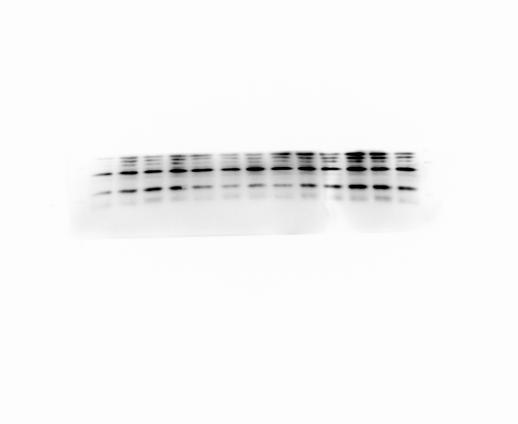


P62


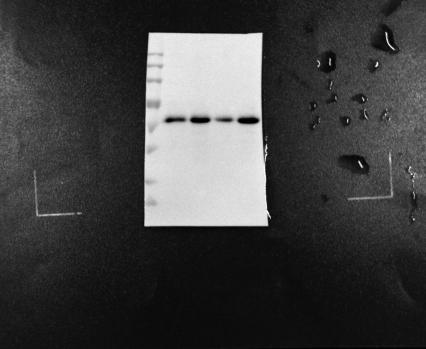


BECLIN1


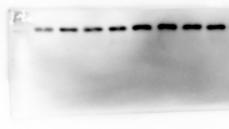


GAPDH


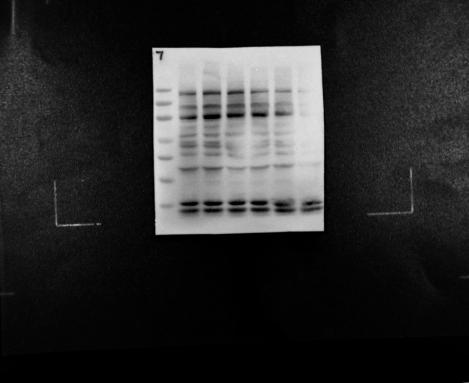


F

Lc3


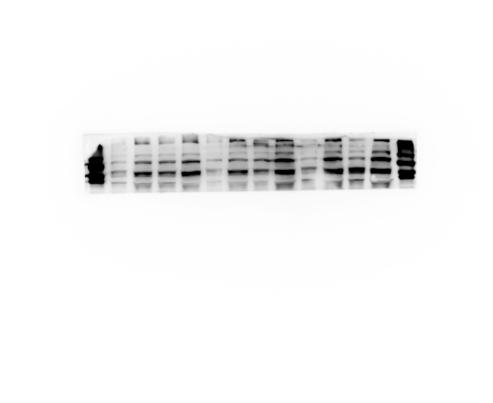


P62


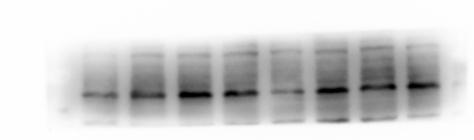


Beclin1


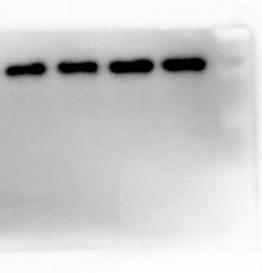


Gapdh


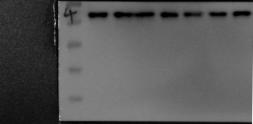


I

VPS34


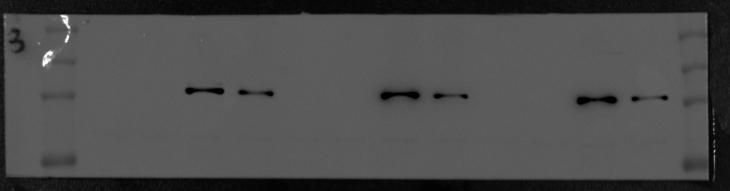


ATG14


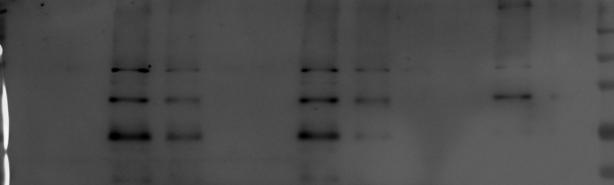


BECLIN1


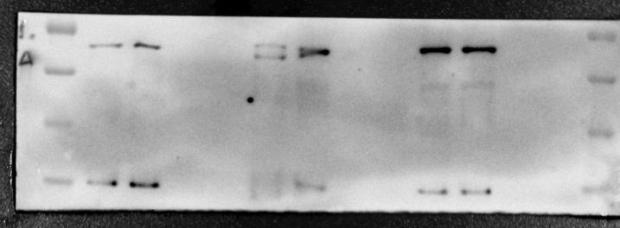


VPS34


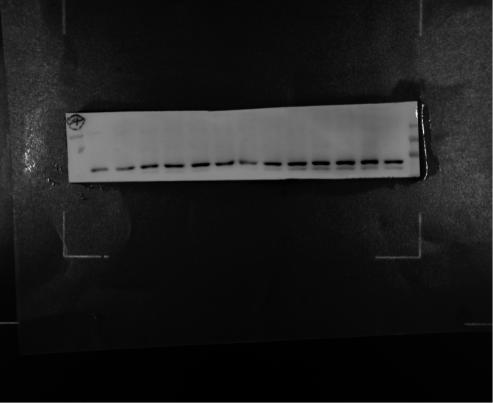


ATG14


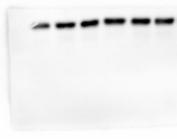


Beclin1


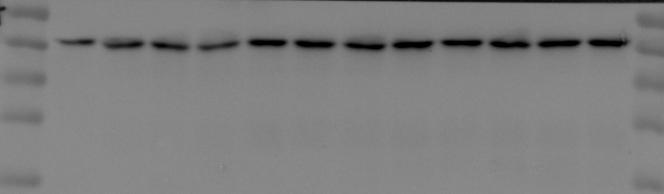


Gapdh


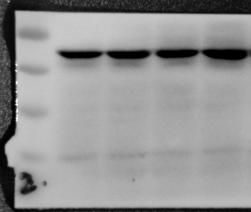


J

PMTOR


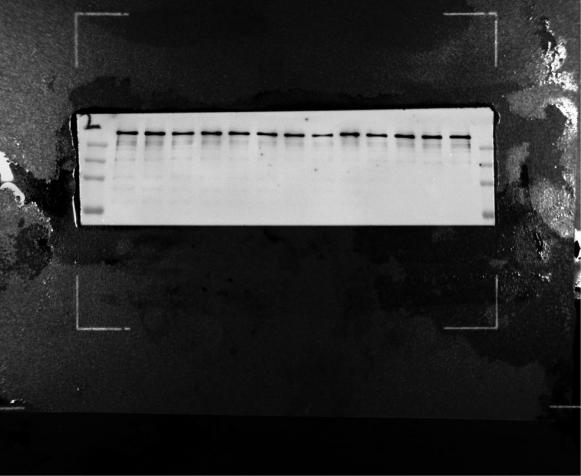


MTOR


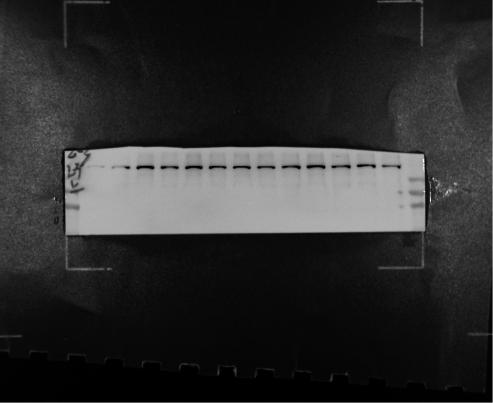


PEIF4EBP1


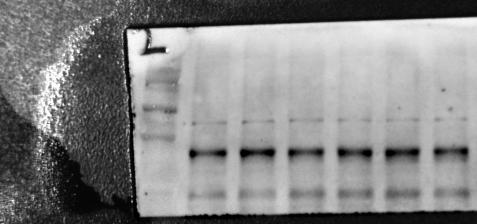


EIF4EBP1


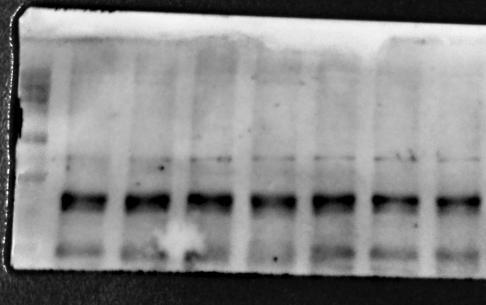


Gapdh


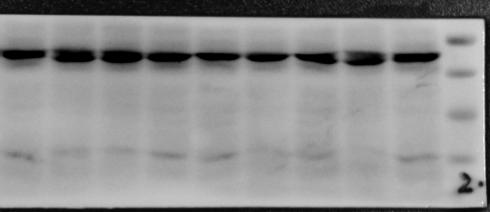


**Fig3**

C

Py


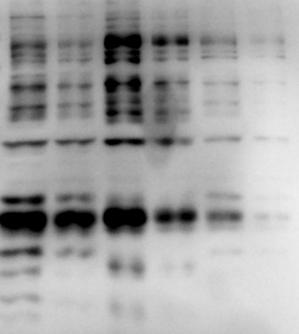


Gapdh


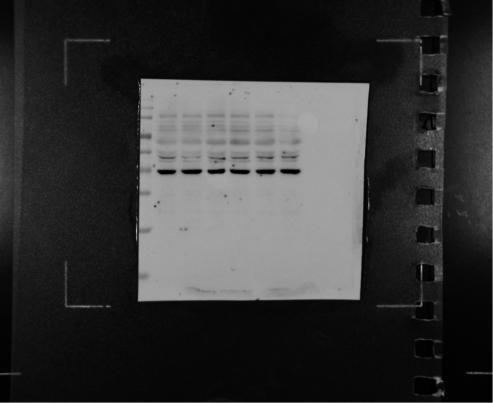


D

Panc1

Pddr1


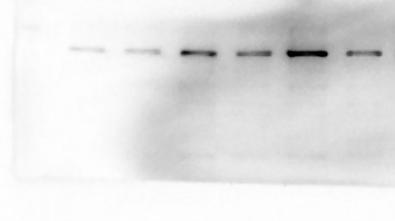


DDR1


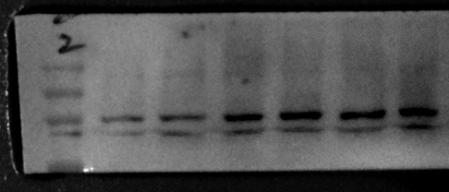


GAODH


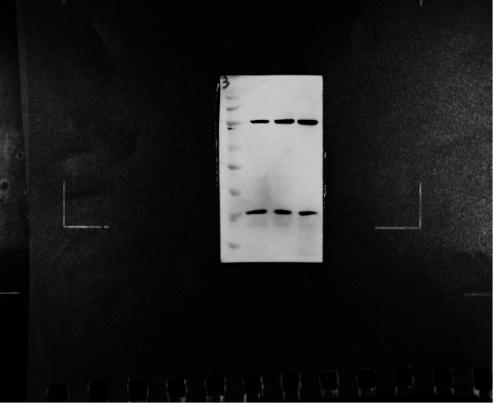


ASPC1

PDDR1


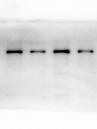


DDR1


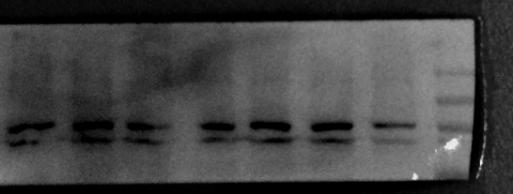


GAPDH


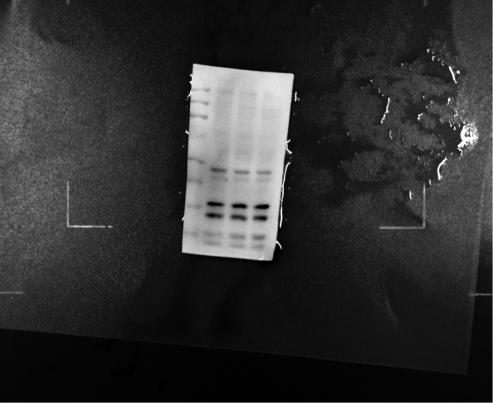


BXPC3

PDDR1


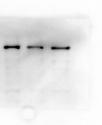


DDR1


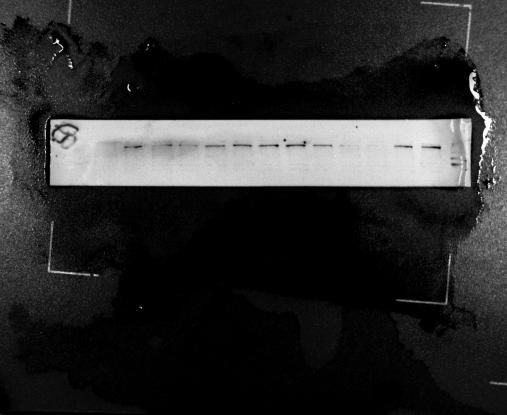


GAPDH


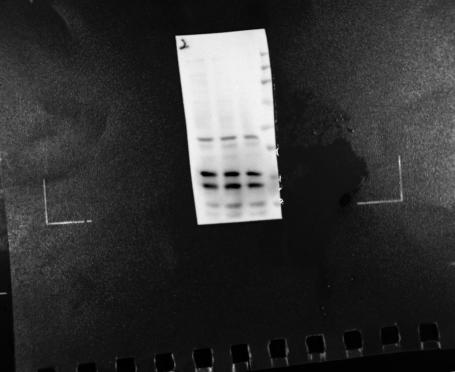


H

Panc1

Pddr1


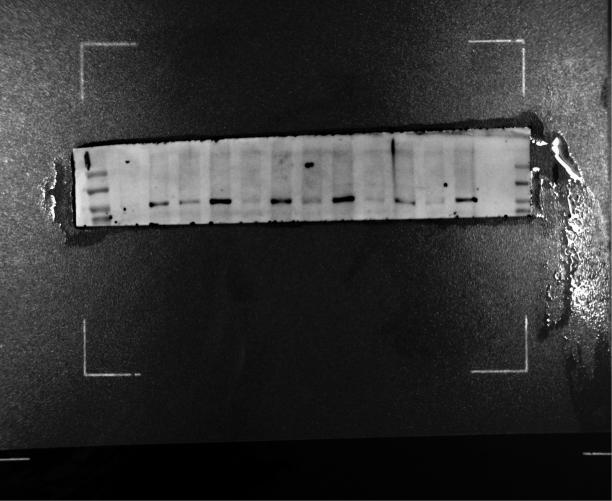


Ddr1


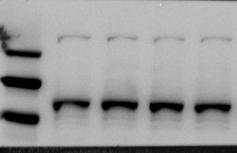


Psrc


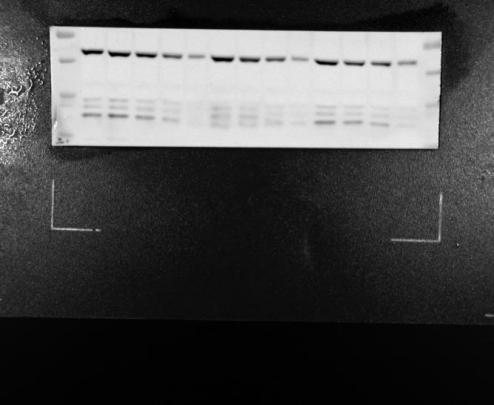


Src


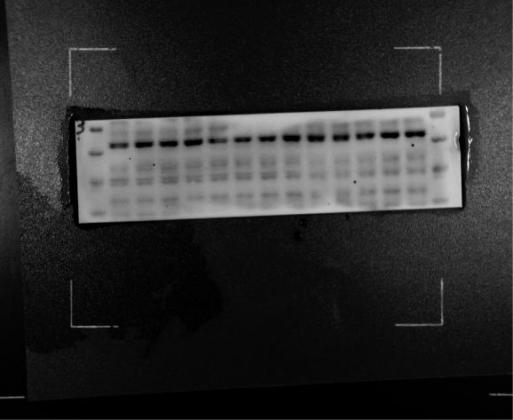


Lc3


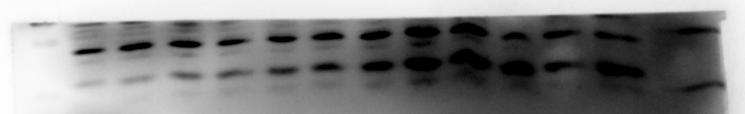


P62


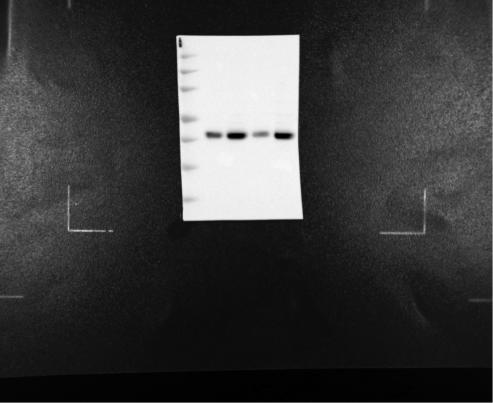


Gapdh


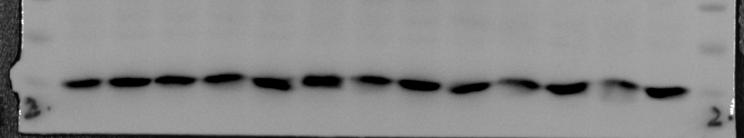


Aspc1

pddr1


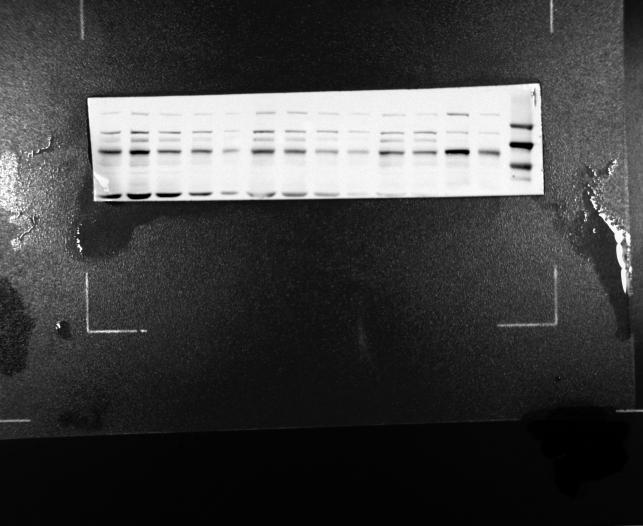


Ddr1


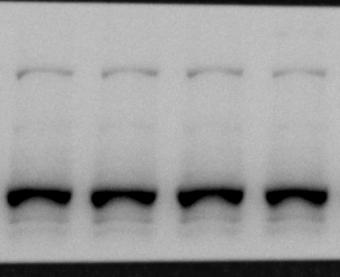


Psrc


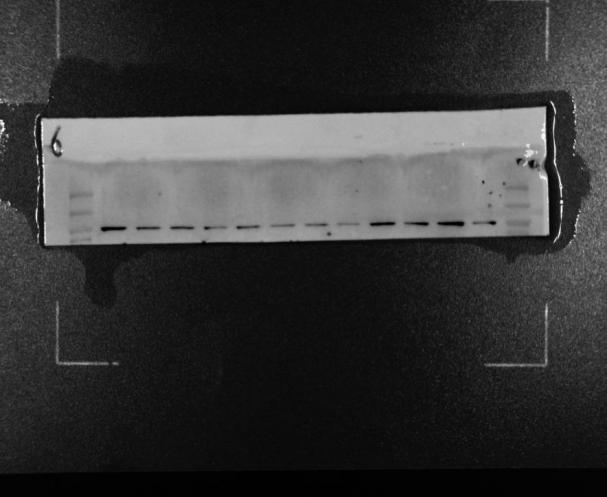


Src


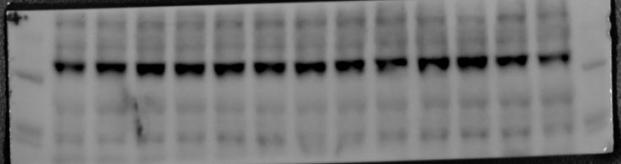


Lc3


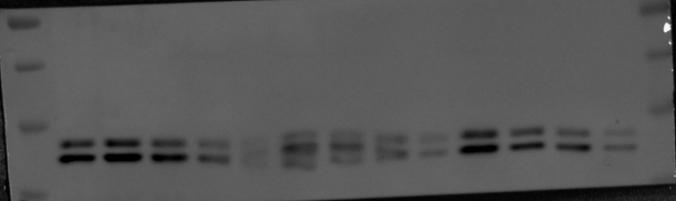


P62


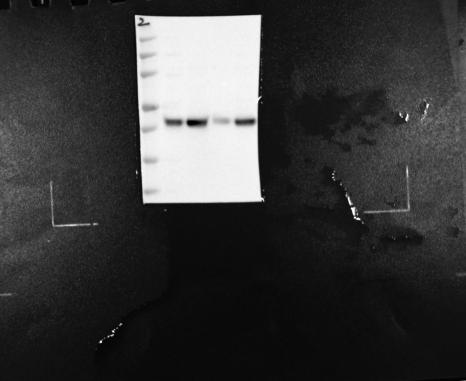


Gapdh


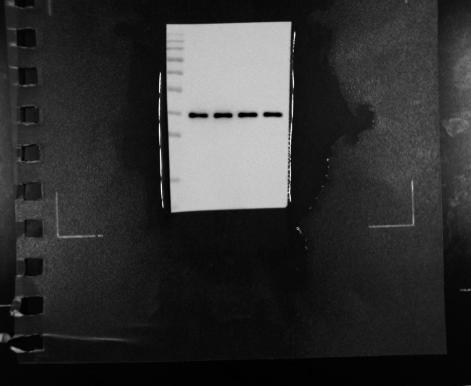


Bxpc3

Pddr1


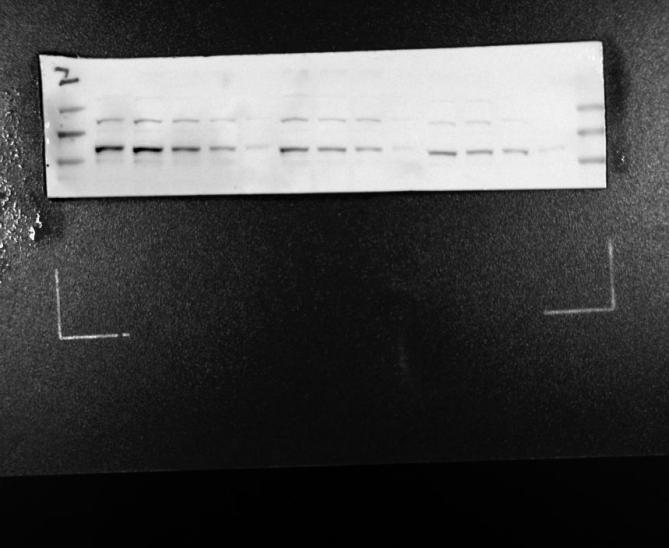


Ddr1


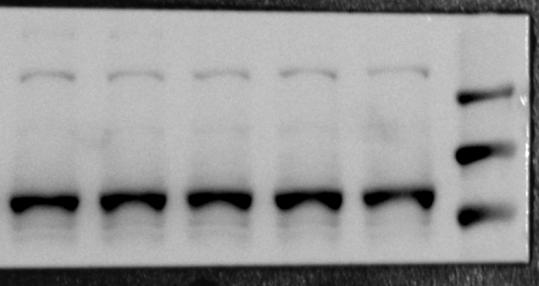


Psrc


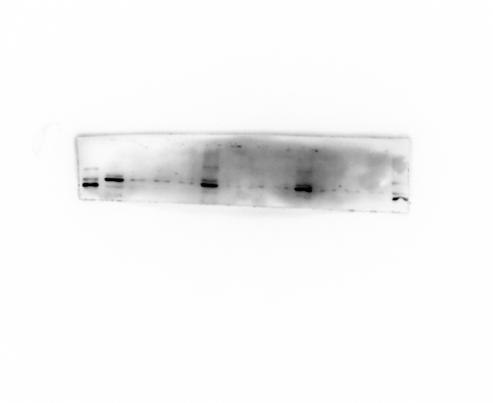


Src


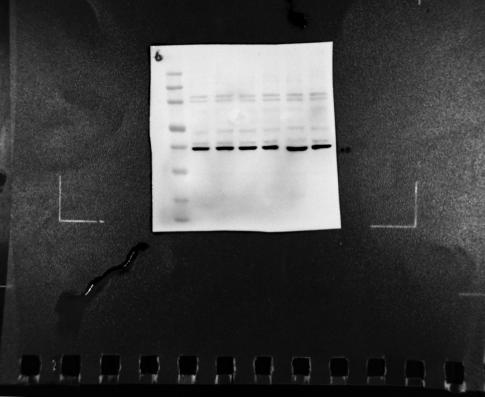


Lc3


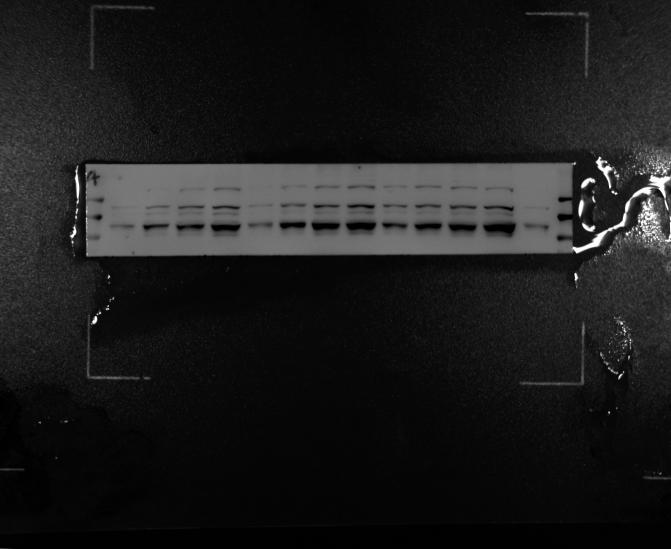


P62


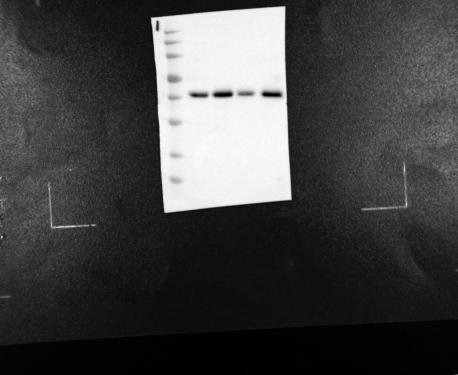


Gapdh


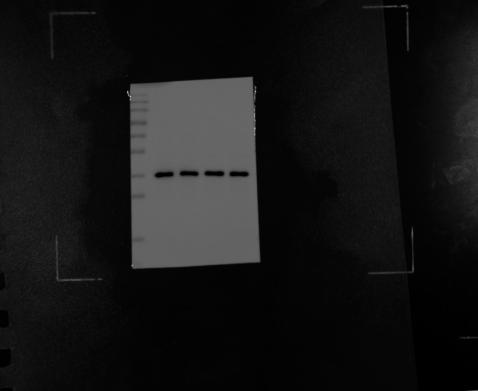


I

PANC1

DDR1


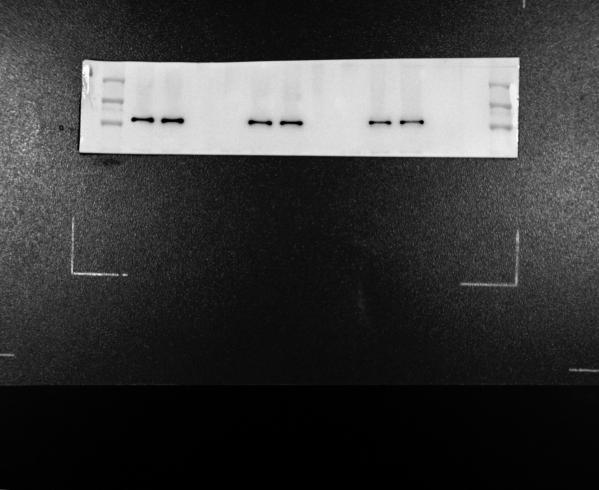


14-3-3


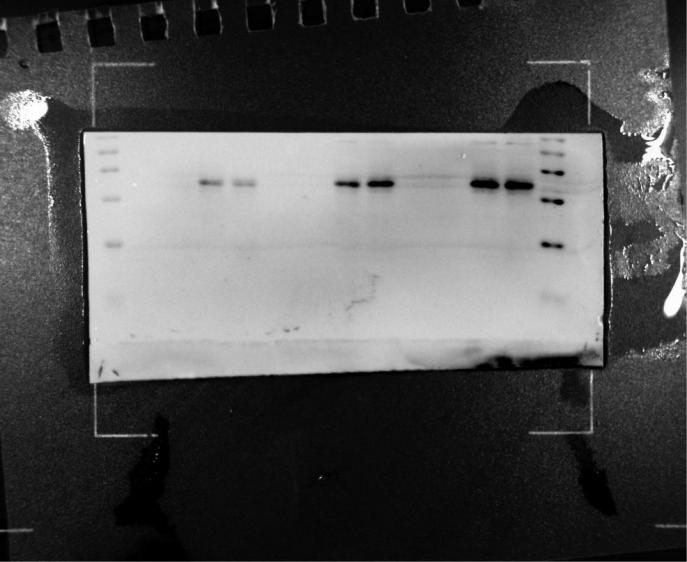


BECLIN1


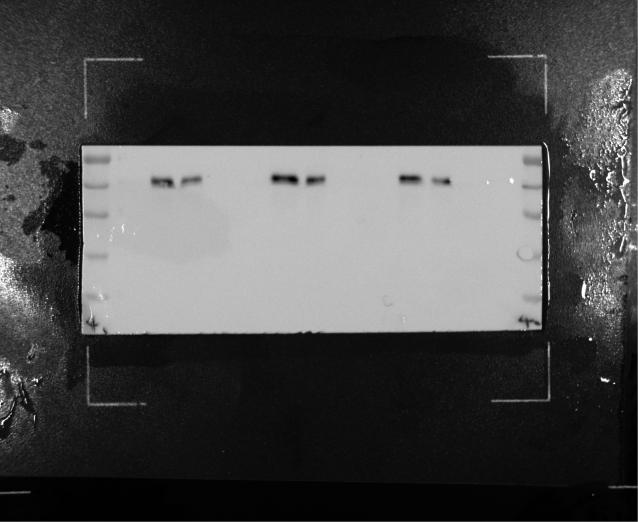


AKT1


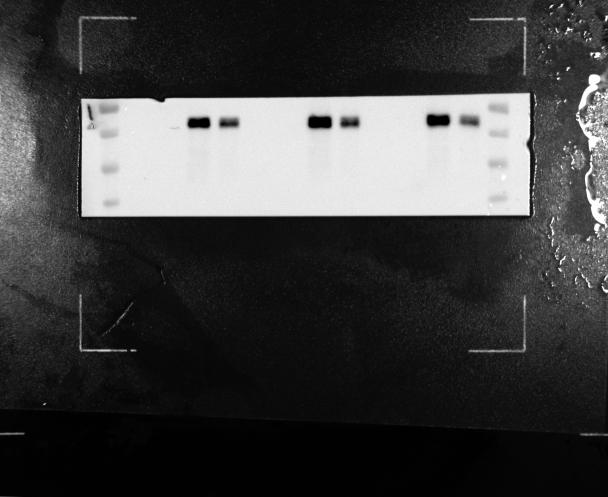


DDR1


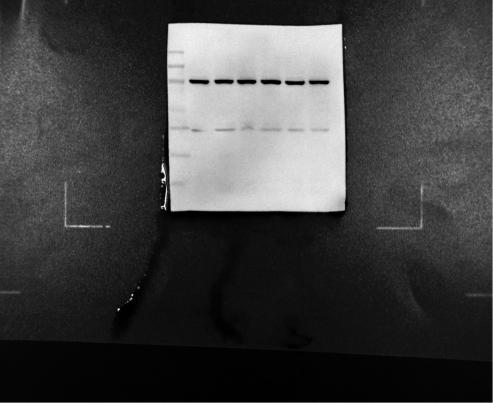


14-3-3


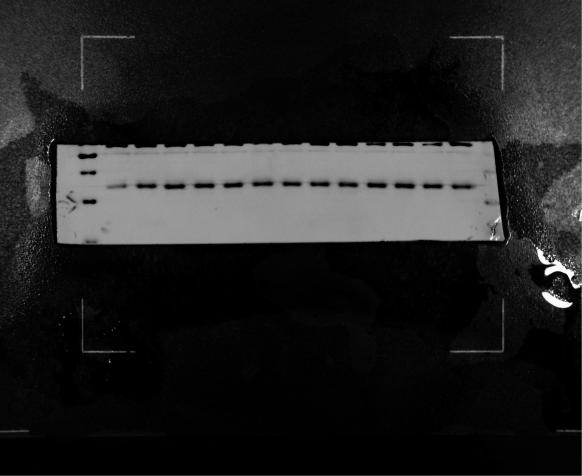


BECLIN1


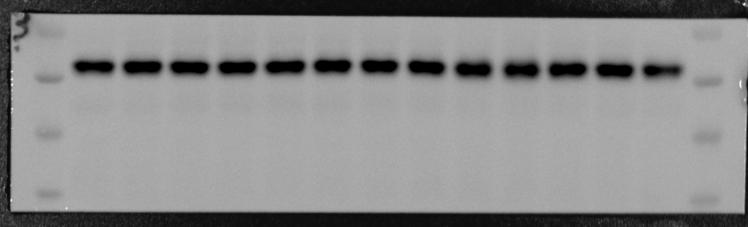


AKT1


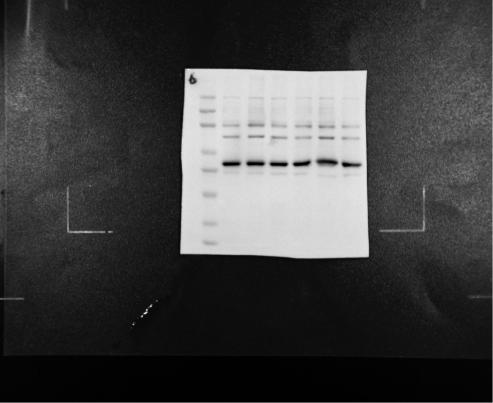


GAPDH


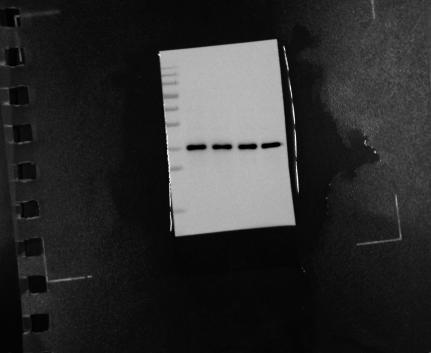


ASPC1

DDR1


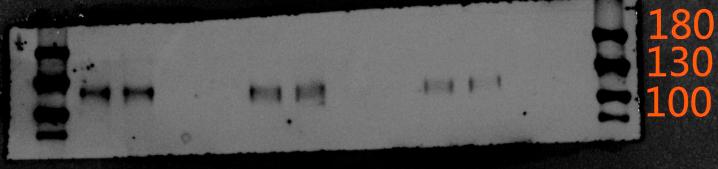


14-3-3


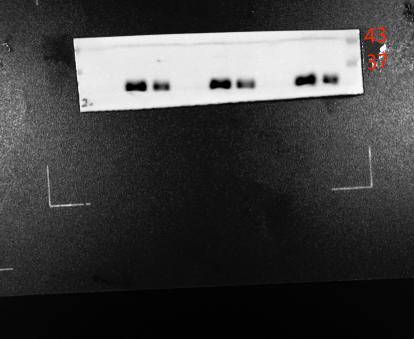


BECLIN1


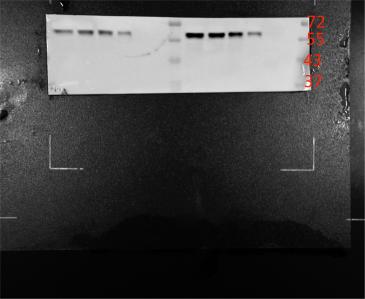


AKT1


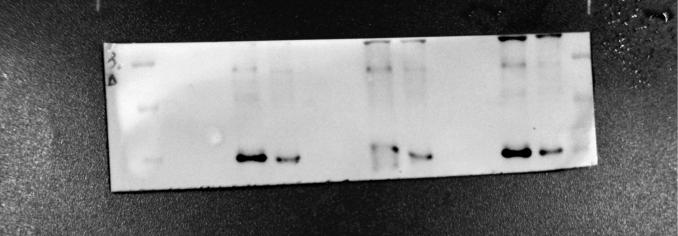


DDR1


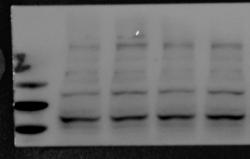


14-3-3


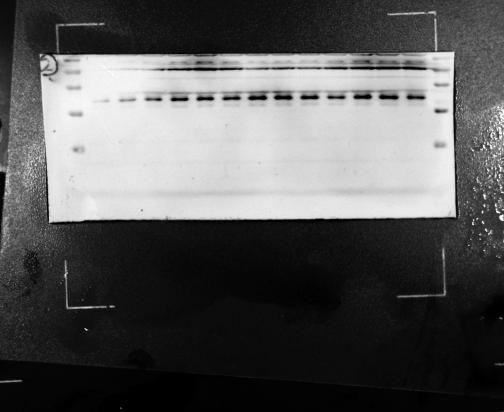


BECLIN1


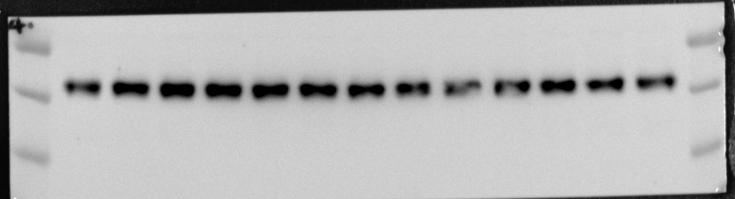


AKT1


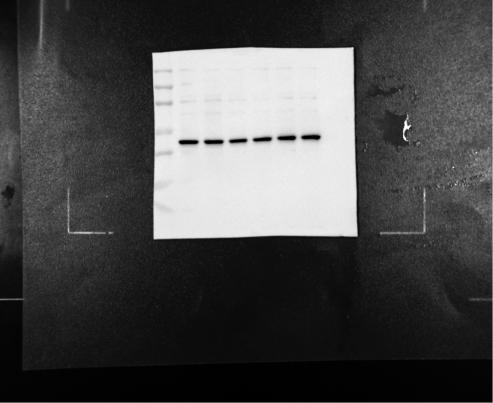


GAPDH

BXPC3

DDR1

14-3-3

BECLIN1

AKT1

DDR1

14-3-3

BECLIN1

AKT1

GAPDH

**FIG4**

A

Pddr1

Ddr1

Gapdh

B

PDDR1

DDR1

PPYK2

PYK2

PERK1/2

ERK1/2

LC3

P62

GAPDH

C

PDDR1

DDR1

GAPDH

D

PDDR1

DDR1

PPYK2

PYK2

PERK1/2

ERK1/2

LC3

P62

GAPDH

E

PDDR1

DDR1

PPYK2

PYK2

PERK1/2

ERK1/2

LC3

P62

GAPDH

H

PDDR1

DDR1

PPYK2

PYK2

PERK1/2

ERK1/2

LC3

P62

GAPDH

I

PDDR1

DDR1

PPYK2

PYK2

PERK1/2

ERK1/2

LC3

P62

GAPDH

J

VAMP8

SNAP29

STX17

VAMP8

SNAP29

STX17

GAPDH

K

VAMP8

SNAP29

STX17

VAMP8

SNAP29

STX17

DDR1

GAPDH

M

LC3

STX17

LC3

STX17

GAPDH

L

Vamp8

Snap29

Stx17

Vamp8

Snap29

Stx17

Flag

Gapdh

**FIG5**

G

PANC1

LC3

P62

GAPDH

PANC1/GEM

LC3

P62

GAPDH

K

PANC1

PDDR1

DDR1

PPYK2

PYK2

PERK1/2

ERK1/2

GAPDH

PANC1/GEM

PDDR1

DDR1

PPYK2

PYK2

PERK1/2

ERK1/2

GAPDH

L

VAMP8

SNAP29

STX17

VAMP8

SNAP29

STX17

GAPDH

FIG6

H

PANC1

LC3

P62

GAPDH

PANC1-GEM

LC3

P62

GAPDH

I

PANC1

PMTOR

MTOR

PEIF4EBP1

EIF4EBP1

PDDR1

DDR1

PPYK2

PYK2

PERK1/2

ERK1/2

PY

GAPDH

PANC1-GEM

PMTOR

MTOR

PEIF4EBP1

EIF4EBP1

PDDR1

DDR1

PPYK2

PYK2

PERK1/2

ERK1/2

PY

GAPDH

FIG S2 new

G

Panc1

Bax

Bcl2

GAPDH

Aspc1

Bax

Bcl2

GAPDH

Bxpc3

Bax

Bcl2

GAPDH

H

Panc1

Pddr1(y513)

DDR1

PJAK2

Jak2

Pstat3

Stat3

Gapdh

Aspc1

Pddr1(y513)

DDR1

PJAK2

Jak2

Pstat3

Stat3

Gapdh

Bxpc3

Pddr1(y513)

DDR1

PJAK2

Jak2

Pstat3

Stat3

Gapdh

**I**

**Lc3**

**P62**

**Gapdh**

FIGS3 new

A

aspc1

PDDR1

DDR1

PPYK2

PYK2

PERK1/2

ERK1/2

LC3

P62

GAPDH

C

PDDR1

DDR1

PPYK2

PYK2

PERK1/2

ERK1/2

LC3

P62

GAPDH

FIG S4 new

C

NRF2

KEAP1

GAPDH

FIG S5 new

A

PDDR1

DDR1

PPYK2

PYK2

PERK1/2

ERK1/2

GAPDH

B

PDDR1

DDR1

PPYK2

PYK2

PERK1/2

ERK1/2

GAPDH

C

P-gp

GAPDH
